# How structural variations in synthetic cannabinoids affect their metabolism: A pilot study on the in vivo metabolism of 8 synthetic cannabinoid receptor agonists

**DOI:** 10.1007/s00204-026-04517-6

**Published:** 2026-07-23

**Authors:** María Mata-Pesquera, David Fabregat-Safont, Manuela Barneo-Muñoz, María José Sánchez-Catalán, Ferran Martinez-Garcia, Marie Mardal, Juan V. Sancho, María Ibáñez

**Affiliations:** 1 Environmental and Public Health Analytical Chemistry, Research Institute for Pesticides and Water, University Jaume I, Avda. Sos Baynat s/n, 12071 Castelló de la Plana, Spain; 2 Applied Metabolomics Research Group, Hospital del Mar Research Institute, 88 Doctor Aiguader, 08003 Barcelona, Spain; 3 Research Unit on Functional Neuroanatomy (NeuroFun), Predepartamental Unit of Medicine, Faculty of Health Sciences, University Jaume I, Castelló de la Plana, Spain; 4 Section of Forensic Chemistry, Department of Forensic Medicine, University of Copenhagen, Copenhagen, Denmark

**Keywords:** Synthetic cannabinoids, *In vivo* metabolism, High-resolution mass spectrometry, New psychoactive substances, Bioanalysis

## Abstract

The structural diversity and continuous emergence of synthetic cannabinoid receptor agonists (SCRAs) pose an important challenge to forensic and clinical toxicology. Understanding their metabolic pathways is essential for identifying reliable consumption biomarkers and improving toxicological screening strategies. This pilot study has investigated the influence of chemical structure on the metabolism of eight structurally diverse SCRAs: AMB-FUBINACA, ADB-FUBINACA, SDB-005, APINACA, CUMYL-4CN-BINACA, 5 F-AMB-PINACA, 5 F-AB-PINACA, and THJ-2201. The compounds were administered to male Sprague-Dawley rats from which serum and urine samples were analysed using liquid chromatography coupled to high-resolution mass spectrometry (LC-HRMS). Metabolite identification was achieved through different analytical strategies. Following structural elucidation, the in vivo metabolism for each SCRA was evaluated, and the corresponding metabolic pathways were proposed. A comparative analysis of the different metabolic reactions was subsequently performed, classifying biotransformations according to the site of occurrence within the molecule, to highlight both shared features and compound-specific pathways. The results demonstrated significant variability in the number and type of metabolites identified, underscoring the substantial influence of chemical structure on SCRA metabolism. Despite this variability, consistent biotransformations across compounds were also observed. Notably, this in vivo study allowed the detection of several metabolites not previously reported in in vitro models, demonstrating the added value of in vivo approaches in profiling the complete metabolic fate of SCRAs.

**Supplementary Information:**

The online version contains supplementary material available at https://doi.org/10.1007/s00204-026-04517-6.

## Introduction

**Fig. 1:**
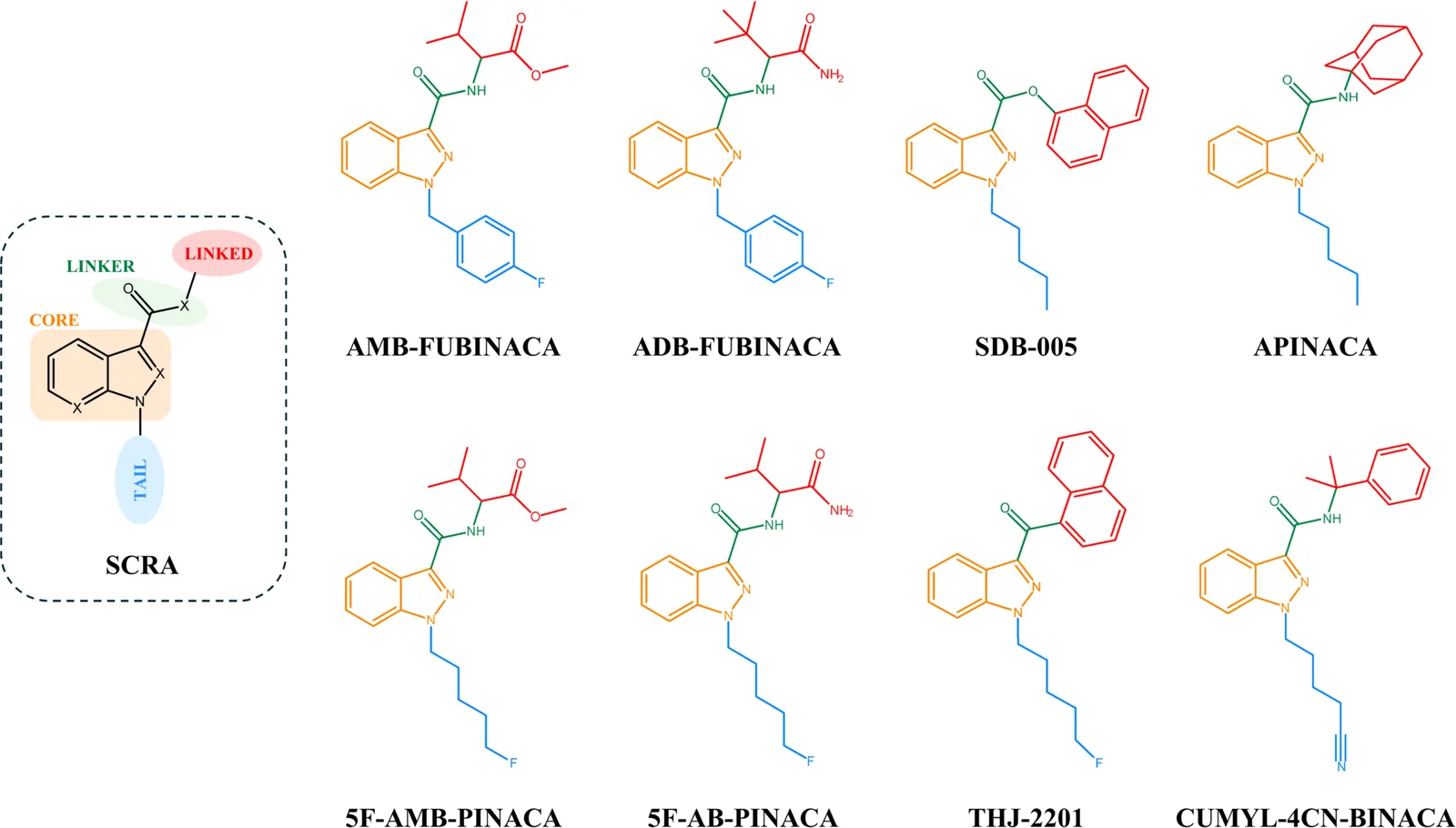
General SCRA structure and structures of the SCRAs included in this study

The widespread availability of synthetic cannabinoid receptor agonists (SCRAs) in the recreational market of new psychoactive substances (NPS) (Lobato-Freitas et al. 2021) has become a significant public health concern due to their potential for abuse, and the risk of severe adverse effects, including acute intoxications or even fatalities (Adamowicz et al. 2019; Barceló et al. 2017; Simon et al. 2023). More than 270 SCRAs have been reported to date (EUDA, 2025), most of them sharing a common structural design based on four building blocks: a core, a tail, a linker, and a linked group (see Fig. 1) (Mata-Pesquera et al. 2024). The core group typically consists of an aromatic ring, while the tail is an alkyl chain, often substituted with a halogen atom. The linker acts as a bridge connecting the core to the linked group, which may be either aromatic or non-aromatic. Illicit manufacturers exploit this modularity by constantly modifying and recombining these subunits, generating structurally diverse SCRAs that often evade regulatory controls (Mata-Pesquera et al. 2024; Potts et al. 2020).

This great diversity presents a significant challenge for both analytical chemists and toxicologists. The rapid emergence of novel SCRAs requires constant update of analytical methods for their detection and monitoring (Diao et al. 2019). The problem becomes even more pronounced when attempting to identify SCRAs in intoxication cases, as their rapid and extensive metabolism combined with high potency often results in a complete disappearance or undetectability of the parent compound in urine and blood (Ibáñez et al. 2024; Baginski et al. 2025). Therefore, metabolism studies are essential to identify suitable metabolites which can be used for detecting SCRAs consumption (Diao et al. 2017). Furthermore, elucidating SCRA metabolic pathways can provide valuable insights into their metabolic fate, pharmacokinetics and even toxicity, since each metabolite may interact differently with cannabinoid receptors.

Ideally, metabolism studies would rely on samples from human volunteers or authentic consumer cases. However, ethical concerns and limited access to certain biological matrices, particularly tissues, constrain this approach. Additionally, human data are often derived from intoxication cases involving unknown dosages, polydrug consumption, and variable sampling intervals, all of which may complicate the interpretation of metabolic pathways. In this context, in vivo animal models offer a controlled environment to systematically generate and characterize these biomarkers. Although comprehensive pharmacokinetic studies based on time-resolved sampling would provide a more detailed evaluation of temporal metabolite distribution (Fabregat-Safont et al. 2022), such designs require a significantly larger number of animals. Therefore, as a pragmatic and ethical compromise, single time-point studies can provide a representative ‘snapshot’ of the in vivo metabolic fate of these compounds, allowing for the identification of the most relevant diagnostic biomarkers under controlled conditions.

In this research, the metabolism of eight SCRAs (AMB-FUBINACA, ADB-FUBINACA, SDB-005, APINACA, CUMYL-4CN-BINACA, 5 F-AMB-PINACA, 5 F-AB-PINACA, and THJ-2201) (see Fig. 1; Table 1) has been studied in serum and urine of male Sprague-Dawley rats following intraperitoneal administration. While the compounds selected for this study are not among the most recently emerged SCRAs, they represent structurally diverse families that maintain a high epidemiological prevalence in forensic toxicology. Additionally, although the intraperitoneal route of administration does not fully mimic the inhalation route commonly associated with human consumption, it is widely used in controlled metabolism studies and is expected to yield comparable metabolic pathways. Analyses were performed using liquid chromatography coupled to high-resolution mass spectrometry (LC-HRMS). To our knowledge, this represents the first in vivo metabolic characterization of these compounds in rats, except for AMB-FUBINACA, which has been previously investigated (Fabregat-Safont et al. 2022). Metabolite identification was achieved through different analytical strategies. Subsequent comparison of biotransformations allowed to highlight both shared features and distinct aspects of their metabolic pathways.

**Table 1:** Structural components (tail, core, linker and linked groups) of the eight selected SCRAs

SCRA	TAIL	CORE	LINKER	LINKED GROUP
	4-Fluorobenzyl	Indazole	Carboxamide	Methyl-3-methyl-butanoate
ADB-FUBINACA	4-Fluorobenzyl	Indazole	Carboxamide	3,3-Dimethyl-butanamide
SDB-005	Pentyl	Indazole	Carboxylate	Naphthyl
APINACA	Pentyl	Indazole	Carboxamide	Adamantyl
CUMYL-4CN-BINACA	4-Cyanobutyl	Indazole	Carboxamide	Cumyl
5 F-AMB-PINACA	5-Fluoropentyl	Indazole	Carboxamide	Methyl-3-methyl-butanoate
5 F-AB-PINACA	5-Fluoropentyl	Indazole	Carboxamide	3-Methyl-butanamide
THJ-2201	5-Fluoropentyl	Indazole	Methanone	Naphthyl

## Materials and methods

### Reagents and chemicals

SCRAs analytical standards were kindly provided and purity tested by the Slovenian National Forensic Laboratory. SCRAs stock solutions were prepared at approximately 1 mg/mL in methanol (0.01 mg accuracy).

Ultrapure water was obtained by purifying demineralised water using an Ultramatic Plus GR from Wasserlab (Navarra, Spain). LC-MS grade acetonitrile (ACN), methanol (MeOH), sodium hydroxide (NaOH), formic acid (HCOOH), TWEEN80 and ethanol were purchased from Scharlau (Scharlab, Barcelona, Spain). Leucine enkephalin acetate salt hydrated (> 95%) was purchased from Merck (Darmstadt, Germany). Physiological saline solution was acquired from Laboratorios ERN (Barcelona, Spain).

### Animal experiments

The study was approved by the Committee of Ethics and Animal Experimentation of the Universitat Jaume I and the Conselleria de Agricultura de la Generalitat Valenciana (Spain; reference 2019/VSC/PEA/048), in accordance with the European Directive 2010/63/EU for the protection of animals used for scientific purposes.

Experimental animals were 18 male Sprague-Dawley rats (Janvier Labs, France) aged 8 weeks upon arrival at the animal facilities. Males were habituated to the facilities for at least one week and weighed between 385 and 425 g on the experimental day. They were individually housed in polypropylene plastic cages for one week prior to the experiment (42 × 21 × 20 cm). The rats were kept under controlled conditions, with a temperature of 24 ± 2 °C and a 12-hour light/dark cycle (lights ON at 8 AM) with food and water *ad libitum*.

SCRAs were administered intraperitoneally at a dose of 1 mg/kg, selected based on similar studies reported in the literature (Fabregat-Safont et al. 2022; Carlier et al. 2018). Two rats were used per compound, together with two additional control animals. Although a higher number of animals per compound would have been desirable to better assess inter-individual variability, this was limited by ethical considerations and the application of the principles of the 3Rs (Reduction, Replacement, and Refinement). The injection solution was prepared at 1 mg/mL in physiological saline solution containing 5% ethanol and 5% TWEEN80 as vehicle. Between 385 and 425 µL of that solution were injected depending on animal weight. Additionally, two control animals were administered with the same vehicle solution without any active compound.

Blood and urine samples from each animal were collected 90 min after injection, following anesthesia with CO_2_ and immediate decapitation. Blood was mainly collected from the carotid arteries and jugular veins and kept at room temperature for 30 min to allow coagulation. Then, samples were centrifuged at 2,000 rcf (Biocen 22 R centrifuge, Ortoalresa, Madrid, Spain) for 10 min at 4 °C to separate serum from sedimented clot. Regarding urine samples, they were directly collected from the bottom of the cage during the 0–90 min period post-injection and stored in 2 mL polypropylene tubes. For AMB-FUBINACA, ADB-FUBINACA, CUMYL-4CN-BINACA and SDB-005, urine collection was limited to a single specimen because spontaneous urination did not occur within the experimental timeframe. All samples were snap-frozen in liquid nitrogen after collection and stored at -23 °C until analysis.

### Sample treatment

0.1 mL of serum or urine were mixed with 0.3 mL of acetonitrile containing 1% of formic acid to precipitate proteins. After vortex-mixing for 1 min, samples were frozen for 30 min, centrifuged at 12,500 rfc (Eppendorf 5424R centrifuge, Eppendorf, Hamburg, Germany) for 10 min, and the supernatant was transferred to a vial for UHPLC-HRMS analysis.

### Instrumentation

Samples were analysed using an Acquity ultra-performance liquid chromatography (UPLC) system (Waters Corp, Mildford, MA, USA) coupled to a Xevo G2 QTOF (Waters Corp, Manchester, UK) mass spectrometer with quadrupole time-of-flight (QTOF) hybrid mass analyser. Chromatographic separation was achieved using a Cortecs C18, 100 × 2.1 mm, 2.7 μm particle size analytical column (Waters Corp, Wexford, Ireland). HRMS instrument was operated in the *m/z* 50-1000 range, in both positive and negative ionization modes, using a data-independent acquisition method for detecting precursor and fragment accurate-mass ions. Detailed information about chromatographic separation and HRMS measuring conditions can be found in literature (Mata-Pesquera et al. 2023; Fabregat-Safont et al. 2017, 2018).

### Analytical strategies used for metabolite identification

Three different strategies, satisfactorily applied in previous metabolism experiments (Fabregat-Safont et al. 2022), were used for metabolite detection.

First, a target screening was performed, focusing on metabolites previously described in the literature. In vitro metabolic data (and in vivo data for AMB-FUBINACA) were available for all compounds except for SDB-005 (Fabregat-Safont et al. 2022; Andersson et al. 2016; Öztürk et al. 2018; Stalberga et al. 2023; Gandhi et al. 2013; Kavanagh et al. 2017; Carlier et al. 2017; Wohlfarth et al. 2015; Diao et al. 2016a). Narrow-window Extracted Ion Chromatograms (nw-EIC) were generated at the *m/z* values corresponding to the (de)protonated molecules.

Secondly, a suspect screening approach was applied using the MetaboLynx XS application manager (MassLynx v4.1, Waters Corp.). A list of potential biotransformations was generated based on the structural characteristics of SCRAs, including typical phase I (e.g. oxidation, reduction, hydrolysis…) and phase II (e.g. glucuronidation, sulphation…) reactions. Binary comparisons were performed using the software, allowing the detection of compounds present exclusively in samples from SCRA-dosed animals and absent in control samples, fitting these compounds also with the expected biotransformations of the selected SCRA. Compounds fulfilling both criteria were selected for further evaluation.

Finally, metabolites were investigated using the common fragmentation-based approach (Fabregat-Safont et al. 2022; Ibáñez et al. 2016) taking profit that both parent compound and its metabolites typically share characteristic fragment ions corresponding to unmodified moieties. This strategy was further applied to identify common fragments of previously characterized metabolites (Ibáñez et al. 2016).

Once metabolites were detected using the three strategies described above, their structures were elucidated based on HRMS data. For the first two approaches, the elemental composition of each metabolite was already known. In the third approach, it was determined from the accurate mass and isotopic pattern observed in the low energy (LE) function, enabling the identification of the biotransformation occurred. A mass error tolerance ≤ 5 ppm was established as the primary acceptance criterion. However, slighty higher mass errors were accepted for compounds with lower *m/z* values and very low abundances. To localize the metabolic biotransformation site, the high energy (HE) spectrum of the parent compound was compared with that of the corresponding metabolite. Shared fragments indicated unaltered moieties, while mass differences in fragmentation patterns provided insight into the modified sites.

Metabolite identification confidence levels were assigned according to the framework proposed by Schymanski et al. (2014). Since authentic reference standards were only available for the parent compounds and metabolite M1 of AMB-FUBINACA (Level 1), the identification of metabolites relied on fragmentation spectra interpretation and comparison with previously reported data. Level 2a was assigned to metabolites whose fragmentation patterns matched those described in literature, particularly from prior in vitro studies, providing a high degree of structural certainty. Metabolites not previously described in the literature but exhibiting fragmentation patterns consistent with the proposed structures were classified as Level 2b. When the exact site of a biotransformation could not be unequivocally determined, such in the case of positional hydroxylation isomers, metabolites were assigned to Level 3 to reflect structural ambiguity.

## Results

### Metabolite detection and elucidation

#### First approach: targeted screening

**Fig. 2:**
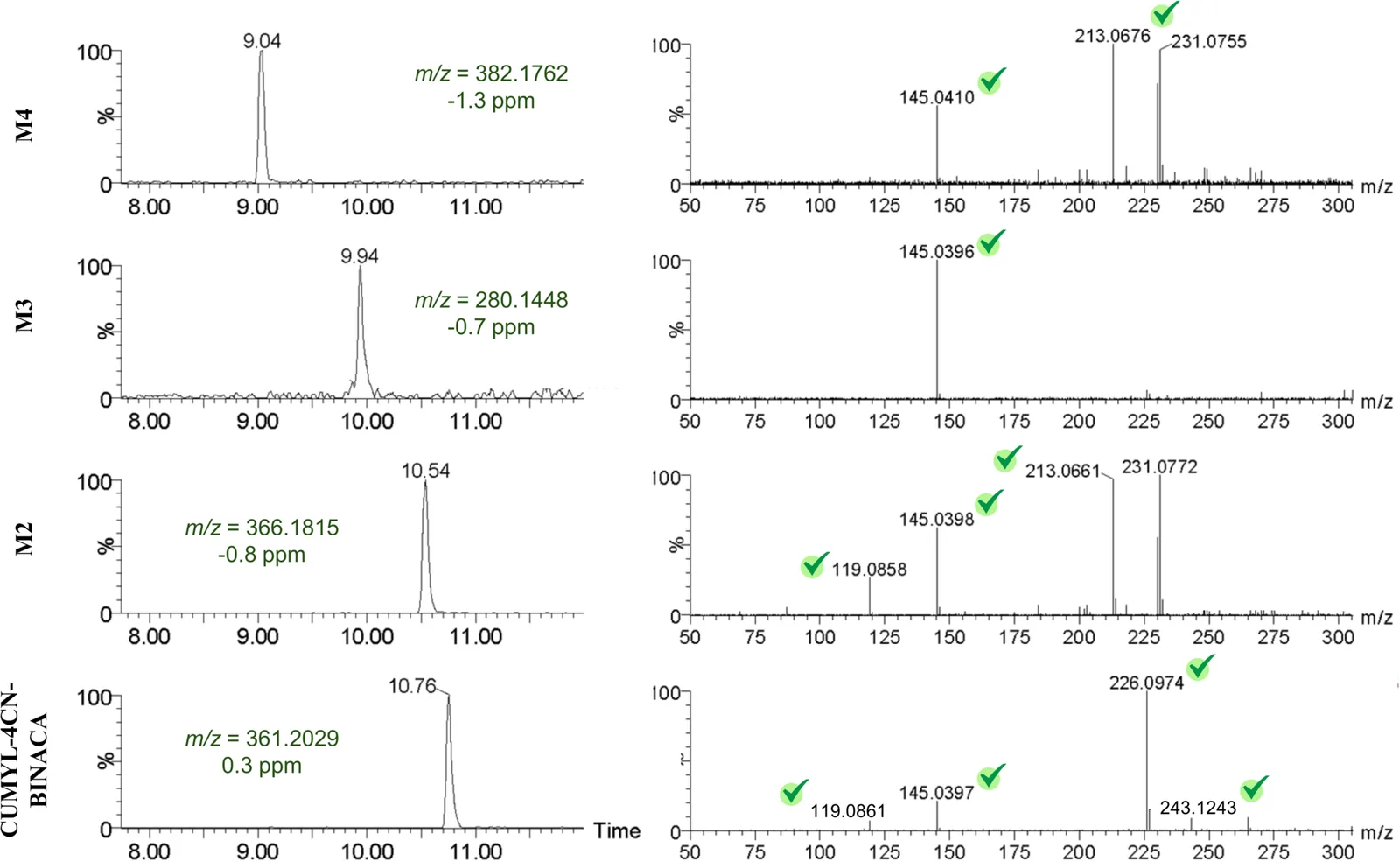
Chromatograms (left) and fragmentation spectra (right) for CUMYL-4CN-BINACA and 3 selected metabolites found in the rat serum of this in vivo study. Green ticks indicate fragment ions that match the previously reported in vitro metabolism data (Öztürk et al. 2018)

The first step for detecting metabolites involved a targeted screening, in which metabolites previously described in the literature were directly searched for. For instance, Fig. 2 shows the nw-EICs and the corresponding fragmentation spectrum for CUMYL-4CN-BINACA and three of its metabolites found in rat serum, which are consistent with previously reported in vitro data. The spectra confirmed their identity, as most of the observed fragment ions matched those reported in the in vitro metabolism study (Öztürk et al. 2018).

#### Second approach: expected biotransformations

**Fig. 3:**
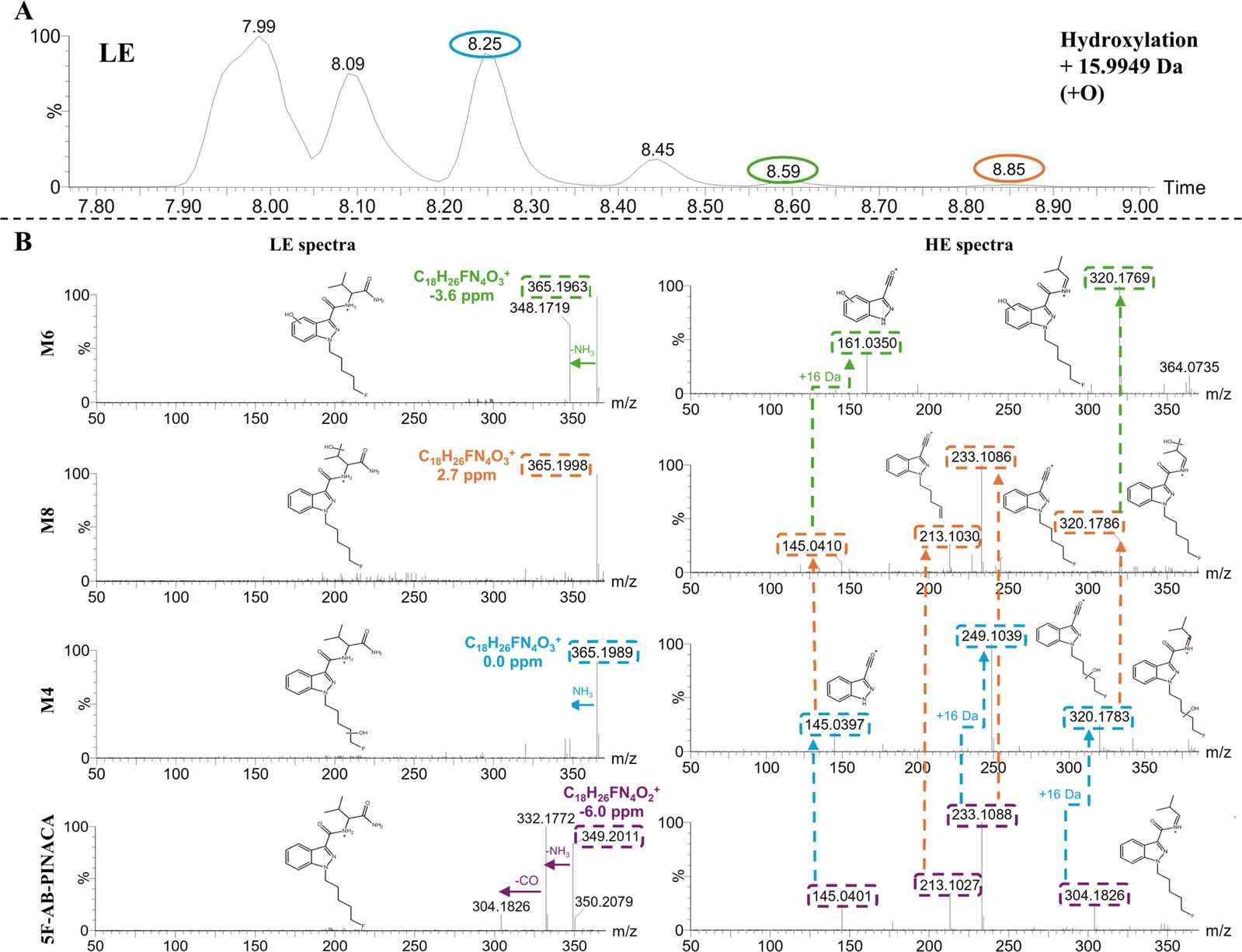
Identification of three hydroxylated 5 F-AB-PINACA metabolites by the expected biotransformation strategy. **A** nw-EIC for *m/z* 365.1985, corresponding to the hydroxylated metabolite in urine. **B** LE (left) and HE (right) spectra for the parent compound and metabolites M4, M8 and M6. Purple text and dashed lines refer to 5 F-AB-PINACA, blue to M4, orange to M8 and green to M6. Mass errors are calculated in parts per million (ppm)

Moving to the next approach, hydroxylation is one of the most common reactions in the metabolism of SCRAs and therefore it was included in the list of expected biotransformations. Figure 3A displays the nw-EIC corresponding to a hydroxylated metabolite of 5 F-AB-PINACA in urine (exact mass *m/z* 365.1989, + 16 Da shift compared to the parent compound) in the 7.8–9 min chromatogram range. The presence of multiple chromatographic peaks at this *m/z* suggests the formation of several hydroxylated metabolites.

As an example, Fig. 3B shows the elucidation of three hydroxylated metabolites eluting at 8.25 min (M4), 8.59 min (M6) and 8.85 min (M8). The LE spectra of these metabolites (Fig. 3B, left) revealed mass errors lower than 4 ppm. The mass shift of + 16 Da was also observed in the HE spectra for the fragment ion at *m/z* 320 (Fig. 3B, right).

For M4 (8.25 min), the fragment ion at *m/z* 249 shows the + 16 Da shift relative to the parent fragment at *m/z* 233, indicating hydroxylation on either the indazole ring or the fluoropentyl chain. The presence of the shared fragment at *m/z* 145 rules out modification on the indazole ring, confirming hydroxylation on the fluoropentyl tail.

In contrast, M8 (8.85 min) exhibits the + 16 Da shift only in the fragment ion at *m/z* 320, with the remaining fragments identical to those of the parent. This suggests that hydroxylation likely occurred in the methyl butanamide moiety, most plausibly within the isopropyl group.

Finally, for M6 (8.59 min), in addition to the fragment at *m/z* 320, only the fragment at *m/z* 161 was observed, showing a + 16 Da shift relative to the parent fragment at *m/z* 145, which corresponds to the core structure. This pattern suggests that hydroxylation took place on the indazole ring.

#### Third approach: common fragmentation

**Fig. 4:**
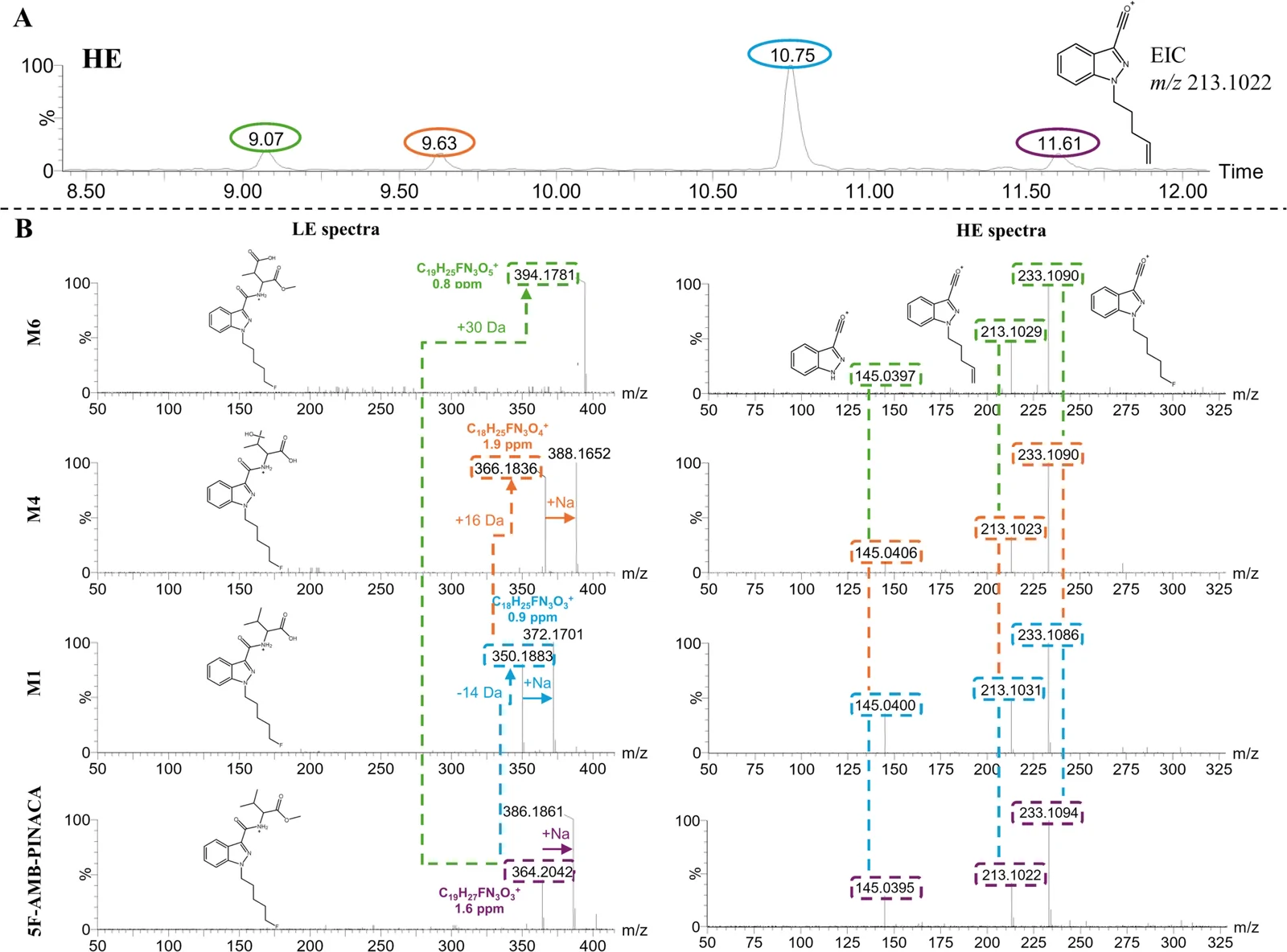
Identification of three 5 F-AMB-PINACA metabolites by common fragmentation pathway. **A** nw-EIC for *m/z* 213.1022, corresponding to a characteristic fragment ion in serum. **B** LE (left) and HE (right) spectra for the parent compound and metabolites M1, M4 and M6. Purple text and dashed lines refer to 5 F-AMB-PINACA, blue to M1, orange to M4 and green to M6. Mass errors are calculated in parts per million (ppm)

SCRAs containing an indazole core and a fluoropentyl tail typically produce a characteristic fragment ion at *m/z* 213.1022. This fragment was therefore employed to identify metabolites following the same fragmentation pathway. Figure 4A shows the nw-EIC of this fragment ion in the serum sample from a rat administered with 5 F-AMB-PINACA. Besides the peak at 11.61 min, corresponding to the parent compound, three additional peaks were observed, indicative of metabolites structurally related to the parent compound.

As shown in Fig. 4B (right), these three metabolites share the same fragmentation spectra. The presence of the fragment ion at *m/z* 233, corresponding to the indazole core with the fluoropentyl tail, indicates that the biotransformation occurred in the linked group. Examination of the LE spectra (Fig. 4B, left) allowed further elucidation.

M1 (eluting at 10.75 min, *m/z* 350.1883, 0.9 ppm) exhibited a mass shift of -14 Da relative to the protonated molecule of 5 F-AMB-PINACA, consistent with an *O*-demethylation. M4 (9.63 min, *m/z* 366.1836, 1.9 ppm) showed a + 16 Da shift relative to M1, indicating hydroxylation of the isopropyl moiety following *O*-demethylation. Finally, M6 (9.07 min, *m/z* 394.1781, 0.8 ppm) presented a + 30 Da shift compared with 5 F-AMB-PINACA, suggesting oxidation to carboxylic acid on the isopropyl moiety.

### In vivo metabolism assessment

Comprehensive information on chromatographic retention times, ionization modes, elemental compositions, accurate-mass fragmentations, and mass errors for all elucidated metabolites is shown in Tables S1-S8. Additionally, they include the relative abundance of each metabolite as well as the corresponding identification confidence level according to the classification proposed by Schymanski et al. (2014). Figures S1-S8 illustrate the proposed metabolic pathways for each of the SCRAs studied. These figures specify the matrix type in which the metabolites were detected and whether they have also been previously reported in in vitro studies (Fabregat-Safont et al. 2022; Andersson et al. 2016; Öztürk et al. 2018; Stalberga et al. 2023; Gandhi et al. 2013; Kavanagh et al. 2017; Carlier et al. 2017; Wohlfarth et al. 2015; Diao et al. 2016a, 2018).

For AMB-FUBINACA, a total of 8 metabolites were detected (Figure S1, Table S1). The predominant metabolite was M1, formed through *O*-demethylation. After *O*-demethylation, hydroxylation on the isopropyl group (M2, M3), lactone formation (M6) and *N*-dealkylation (M8) were also observed. *N*-methylation after *O*-demethylation and *N*-dealkylation yielded M7, a biotransformation previously reported in related studies (Fabregat-Safont et al. 2019, 2022). Additionally, oxidation to carboxylic acid on the isopropyl group produced M4 and M5.

ADB-FUBINACA generated 7 metabolites (Figure S2, Table S2), with hydroxylated (M1) and *N*-dealkylated (M7) metabolites being the most abundant. M1 gave rise to M2 through cyclization, to M3 through *N*-dealkylation and to M4 via amide hydrolysis and cyclization. Subsequent oxidation of M2 produced M5, which underwent *N*-dealkylation to produce M6.

Four metabolites were detected for SDB-005 (Figure S3, Table S3). *O*-denaphtylation yielded M1, for which, curiously, two chromatographic peaks appeared (at 10.54 and 11.0 min) even though there are no chiral or isomerization sites in the structure. Moreover, the retention times of both peaks differ significantly from the SDB-005 standard (13.56 min), indicating that neither corresponds to an in-source fragment of the parent compound. Thus, the exact origin of this phenomenon cannot be unambiguously determined based on the available data and may warrant further investigation. Subsequently, M1 gave rise to an *O*-glucuronide metabolite (M2) and two hydroxylated metabolites on the pentyl tail (M3, M4).

APINACA produced 7 metabolites (Figure S4, Table S4), resulting from one to three hydroxylations on the adamantyl group (M1–M4) or simultaneous hydroxylations on the adamantyl and pentyl groups (M5–M7).

For CUMYL-4CN-BINACA, 4 metabolites were identified (Figure S5, Table S5). M1, M2 and M3 arose from oxidation to carboxylic acid of the cumyl moiety, decyanation to COOH, and *N*-dealkylation, respectively. M4 resulted from cumyl hydroxylation of M2.

5 F-AMB-PINACA presented 22 metabolites (Figure S6, Table S6). The most abundant was the *O*-demethyl metabolite (M1), followed by mono- and di-hydroxylated compounds (M2-M4, M10-M12). Hydroxylations occurred mainly in the isopropyl (linked group) and fluoropentyl (tail) moieties, being also observed in the indazole ring (M19). Additional biotransformations included defluorination to OH (M9) or COOH (M8, M15), *N*-dealkylation (M5, M18, M19), and sulphation (M21, M22), among others.

The compound with the greatest metabolic variety was 5 F-AB-PINACA, with 33 metabolites (Figure S7, Table S7). Predominant modifications included hydroxylation on the fluoropentyl tail (M2-M5), dihydroxylation (M12-M17) and defluorination to OH (M30) or COOH (M28). The remaining metabolites arose from additional hydroxylations (M1, M6-M8), *N*-dealkylation (M9), amide hydrolysis (M10), or combinations thereof (M11, M18-M23). Less common biotransformations, such as epoxide formation followed by hydrolysis to a dihydrodiol (M24), were also observed.

For THJ-2201 (Figure S8, Table S8), the metabolic pathway was simpler, with only 3 metabolites. M1 was formed by defluorination to COOH, M2 via subsequent hydroxylation on the naphthalene moiety, and M3 following a second hydroxylation.

### Comparison between rat in vivo metabolism and reported human data

The metabolic profiles obtained in this study were compared with the available human data to evaluate the translational relevance of the rat in vivo model. Nevertheless, human metabolic data are currently available for only five of the eight compounds investigated herein.

For AMB-FUBINACA, the detected metabolites showed a high degree of concordance with those previously reported in human samples, specifically M1 (*O*-demethylation), which has been described as a key biomarker for quantification in forensic blood samples (Presley et al. 2020).

Similarly, the metabolites identified for ADB-FUBINACA, namely M1 (OH-tert-butyl), M2 (OH-tert-butyl, cyclization) and M5 (OH-tert-butyl, oxidation, cyclization), were consistent with those previously detected in post-mortem blood and urine samples (Kovács et al. 2019; Kavanagh et al. 2017).

In the case of APINACA, the identification of metabolites M1 and M2, corresponding to mono- and di-hydroxylation on the adamantyl moiety, agrees with findings reported in authentic human urine (Vikingsson et al. 2015).

Furthermore, the prevalence of M2 (decyanation to carboxylic acid) for CUMYL-4CN-BINACA is in agreement with its recognition as a major human biomarker in human samples (Öztürk et al. 2018), together with the detection of M4 (decyanation and cumyl hydroxylation).

Finally, for THJ-2201, the detection of M1 (defluorination to COOH) further supports the consistency between rat and human phase I metabolic pathways (Gaunitz et al. 2019).

Although no human data are currently available for the remaining three compounds, the strong agreement observed for the other five SCRAs supports the utility of the rat in vivo model as a valuable approach for metabolic characterization. It not only confirms previously described metabolic routes but also may provide a predictive framework for the identification of biomarkers of emerging SCRAs for which human reference data are still lacking.

## Discussion

**Fig. 5:**
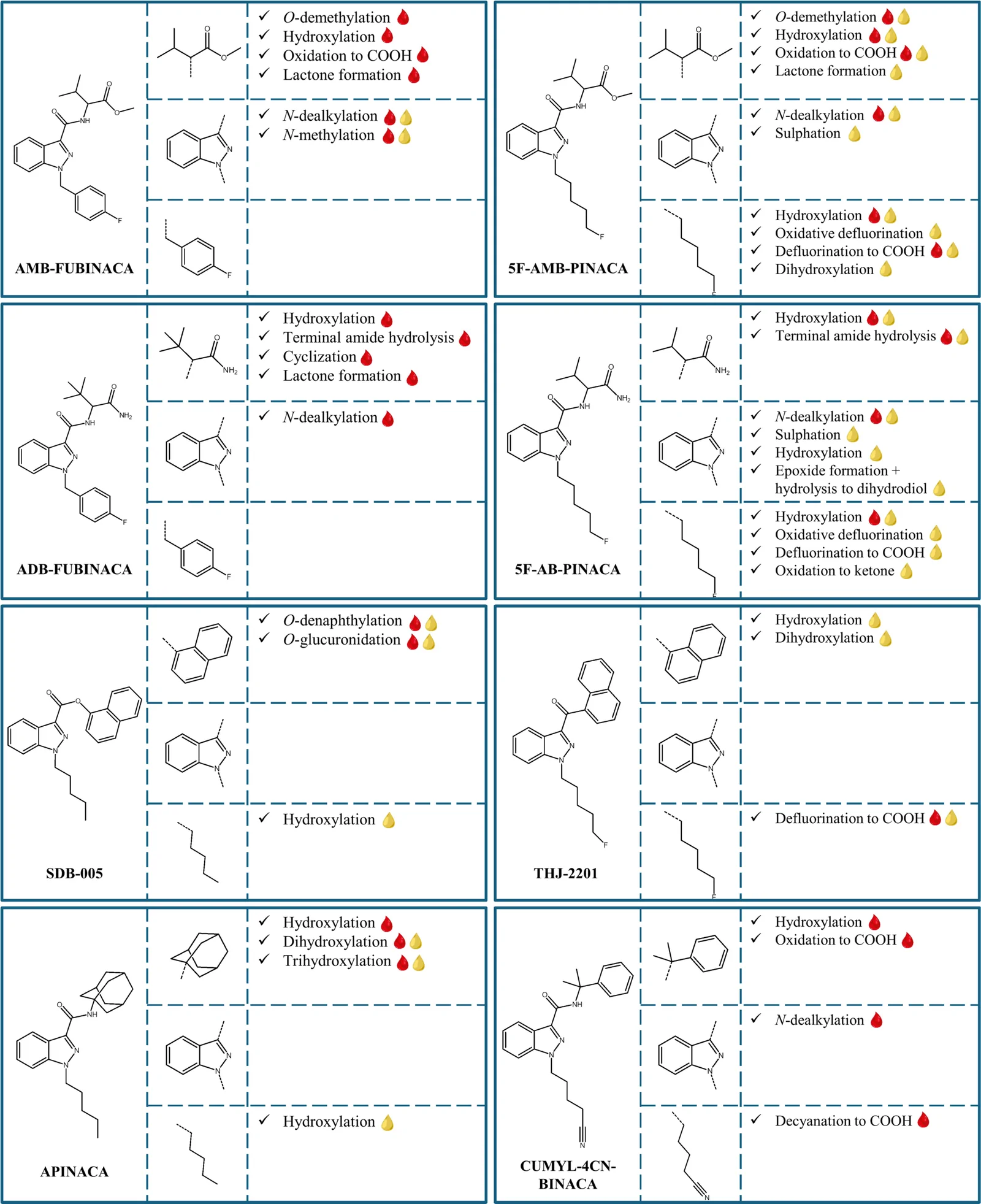
Detailed summary illustrating the distinct site of metabolism and the biotransformations observed for each of the studied compounds. (yellow icons: urine; red icons: blood)

A summary of the observed biotransformations across all compounds is shown in Fig. 5. Despite the considerable structural variety of the SCRAs studied, several common biotransformations were consistently observed. Highlighting these shared metabolic patterns is crucial, as they can guide the identification of biomarkers and facilitate the detection of novel analogues.

The following sections provide a detailed comparison of these metabolic reactions, organized according to the site of occurrence within the molecule. This allows to identify both common and compound-specific pathways and facilitates comparison across different substances. Biotransformations typically occur at the core, the tail and the linked groups of the molecule. On the contrary, the linker moiety exhibits low reactivity, being only involved in the cleavage of the linked group and the formation of phase II metabolites.

### Biotransformations in the core group

As shown in Fig. 1, all SCRAs studied contain an indazole core. Despite this shared core, *N*-dealkylation was observed only for AMB-FUBINACA, ADB-FUBINACA, CUMYL-4CN-BINACA, 5 F-AMB-PINACA and 5 F-AB-PINACA (see Fig. 5). In the case of AMB-FUBINACA, this *N*-dealkylation was followed by *N*-methylation, a biotransformation previously reported in both in vitro and in vivo studies of this compound (Fabregat-Safont et al. 2019, 2022), but not observed in any other SCRA.

Closely related structures, such as 5 F-AMB-PINACA and 5 F-AB-PINACA, which differ only in their linked group, underwent hydroxylation and sulphation on the indazole ring. Nevertheless, the formation of an intermediate epoxide followed by hydrolysis to a dihydrodiol was observed exclusively for 5 F-AB-PINACA, consistent with a previous in vitro study of AB-PINACA metabolism (Wohlfarth et al. 2015).

These findings suggest that biotransformations occurring in the core group are not solely determined by the core structure itself but may also be influenced by other molecular features.

### Biotransformations in the tail group

Hydroxylation was the most common biotransformation observed (see Fig. 5), occurring in compounds with an alkyl chain as tail group (SDB-005, APINACA, 5 F-AMB-PINACA and 5 F-AB-PINACA). In the case of 5 F-AB-PINACA, hydroxylation was further followed by oxidation to a ketone.

One of the most common tail groups in SCRAs is the 5-fluoropentyl chain, present in 5 F-AMB-PINACA, 5 F-AB-PINACA, and THJ-2201. Previous studies indicate that this moiety typically undergoes oxidative defluorination, which can progress to further oxidation, ultimately yielding the corresponding pentanoic acid (Apirakkan et al. 2020; Stalberga et al. 2023). Nevertheless, this metabolic route was not observed for THJ-2201. A similar pathway occurs in compounds with a cyano group at the end of the tail, such as CUMYL-4CN-BINACA, where decyanation is followed by carboxylic acid formation.

Curiously, no biotransformations were observed for SCRAs bearing a 4-fluorobenzyl tail (Fig. 5), consistent with previous studies on similar compounds (Diao et al. 2016b; Watanabe et al. 2023).

### Biotransformations in the linked group

The linked group of SCRAs is typically the main site of metabolism. Hydroxylation of this moiety was observed in all studied SCRAs except for SDB-005, with some compounds, such as APINACA and THJ-2201, undergoing even di- and trihydroxylations (Fig. 5). Other biotransformations within the linked group varied significantly depending on its specific structure, reflecting the influence of structural diversity on metabolic pathways.

Common reactions among compounds sharing methyl-3-methyl-butanoate linked group (AMB-FUBINACA and 5 F-AMB-PINACA) included *O*-demethylation, subsequent hydroxylation of the isopropyl group, lactone formation, and oxidation to carboxylic acid at one or both methyl groups of the isopropyl moiety. Interestingly, the oxidation to carboxylic acid was not limited to this group; for example, it also occurred at one of the methyl groups of the cumyl moiety in CUMYL-4CN-BINACA.

Certain biotransformations appeared to be specific to particular linked groups. For instance, the presence of an additional methyl in the linked group of ADB-FUBINACA with respect to 5 F-AB-PINACA promoted cyclization following hydroxylation, regardless of amide hydrolysis.

Phase II metabolic reactions were also observed in the linked group, such as *O*-glucuronidation of SDB-005.

## Conclusions

This work provides the first in vivo metabolism data in rat for seven of the eight SCRAs included in this pilot study. The number of identified metabolites varied considerably across the compounds studied. 5 F-AB-PINACA and 5 F-AMB-PINACA exhibited the highest metabolic diversity, with 33 and 22 metabolites identified, respectively. In contrast, 8 metabolites were identified for AMB-FUBINACA, while ADB-FUBINACA and APINACA had 7 each. Only 4 metabolites were identified for both CUMYL-4CN-BINACA and SDB-005, and 3 for THJ-2201. The selection of the 90-minute time point was considered suitable as a compromise to capture both ongoing phase I metabolism and early phase II conjugation processes under controlled conditions. However, SCRA pharmacokinetics can vary considerably between compounds, and late-forming metabolites, particularly phase II conjugates such as glucuronides and sulphate derivatives, may not be fully represented at this sampling point. This limitation may be more relevant for compounds exhibiting slower clearance or extensive phase II metabolism. Overall, these findings emphasize the influence of chemical structures in shaping metabolic pathways and support the use of metabolite profiling for SCRA detection. In addition, the combination of previous in vitro data with the present in vivo results is particularly valuable, since metabolites detected under both conditions represent strong candidates for identification in authentic human samples.

In conclusion, this study has identified common metabolic patterns among structurally related SCRAs to support the interpretation of future SCRAs studies. The results obtained highlighted consistent biotransformations across structurally related compounds, as well as shared metabolic behaviors among different chemical structures. Nevertheless, this pilot study has also shown that establishing clear structure-metabolism correlations are not straightforward in certain cases, underscoring the complexity of SCRAs metabolism and complicating the associated public health challenge.

## Supplementary Information

Below is the link to the electronic supplementary material.

Supplementary Material 1
