# Supplementary material for: How structural variations in synthetic cannabinoids affect their metabolism: A pilot study on the in vivo metabolism of 8 synthetic cannabinoid receptor agonists
Source: Arch Toxicol. 2026 Jul 23;100(12):5469–80. doi: 10.1007/s00204-026-04517-6 (PMC13748037; doi:10.1007/s00204-026-04517-6)
Supplement: Supplementary file 1 [file 204_2026_4517_MOESM1_ESM.docx]

**How structural variations in synthetic cannabinoids affect their metabolism? A pilot study on the *in vivo* metabolism of 8 synthetic cannabinoid receptor agonists**

María Mata-Pesquera^1^, David Fabregat-Safont^2,1^, Manuela Barneo-Muñoz^3^, María José Sánchez-Catalán^3^, Ferran Martinez-Garcia^3^, Marie Mardal^4^, Juan V. Sancho^1^, María Ibáñez^1*^

^1^Environmental and Public Health Analytical Chemistry, Research Institute for Pesticides and Water, University Jaume I, Avda. Sos Baynat s/n, 12071 Castelló de la Plana, Spain.

^2^Applied Metabolomics Research Group, Hospital del Mar Research Institute, 88 Doctor Aiguader, 08003 Barcelona, Spain.

^3^Research Unit on Functional Neuroanatomy (NeuroFun) – Predepartamental Unit of Medicine, Faculty of Health Sciences, University Jaume I, Castelló de la Plana, Spain.

^4^Section of Forensic Chemistry, Department of Forensic Medicine, University of Copenhagen, Copenhagen, Denmark.

*Correspondence and request for materials should be addressed to M.I. (email: ibanezm@uji.es).

Tables S1–S8 summarize the metabolites identified for each SCRA, including retention time (RT), accurate mass (*m/z*), ionization mode (ESI), matrix in which the metabolite has been detected, proposed elemental composition, mass error (ppm), characteristic fragment ions, and their corresponding elemental compositions and mass errors. Each metabolite is further annotated with its identification confidence level according to the Schymanski et al. (2014) classification framework. The relative signal intensity for each metabolite is also indicated, assuming comparable ionization efficiencies among compounds.

|  | **Table S1.** UHPLC-HRMS detection of AMB-FUBINACA and its metabolites. | | | | | | | | | | |  |  |
| --- | --- | --- | --- | --- | --- | --- | --- | --- | --- | --- | --- | --- | --- |
| **Compound*** | | **RT (min)** | ***m/z*** | **ESI** | **Matrix (prevalence)** | **Relative area** | **Elemental composition** | **Mass error (ppm)** | **Fragment ions (*m/z*)** | **Elemental composition** | **Mass error (ppm)** | | |
| AMB-FUBINACA^1^ | | 12.02 | 384.1729 | ESIpos | Serum (1/2) | 0.011 | C_21_H_23_FN_3_O_3_^+^ | 1.6 | 253.0774 | C_15_H_10_FN_2_O^+^ | -1.2 | | |
| M1^1^ | | 11.21 | 370.1564 | ESIpos | Serum (2/2) | 1 | C_20_H_21_FN_3_O_3_^+^ | -0.8 | 324.1512 | C_19_H_19_FN_3_O^+^ | 0 | | |
| (*O*-demethyl) | |  |  |  |  |  |  |  | 253.0775 | C_15_H_10_FN_2_O^+^ | -0.8 | | |
|  | |  |  |  |  |  |  |  | 109.0452 | C_7_H_6_F^+^ | -1.8 | | |
|  | |  | 368.1412 | ESIneg | Serum (2/2) | 1 | C_20_H_19_FN_3_O_3_^-^ | 0.5 | 225.0805 | C_14_H_10_FN_2_^-^ | -10.2 | | |
|  | |  |  |  |  |  |  |  | 215.1040 | C_12_H_13_N_3_O^-^ | -8.8 | | |
|  | |  |  |  |  |  |  |  | 117.0448 | C_7_H_5_N_2_^-^ | -4.3 | | |
| M2^2a^ | | 9.46 | 386.1521 | ESIpos | Serum (2/2) | 0.006 | C_20_H_21_FN_3_O_4_^+^ | 1.3 | 253.0785 | C_15_H_10_FN_2_O^+^ | 4.7 | | |
| (*O*-demethyl, | |  |  |  |  |  |  |  | 109.0465 | C_7_H_6_F^+^ | 10.1 | | |
| OH-isopropyl) | |  | 384.1348 | ESIneg | Serum (2/2) | 0.007 | C_20_H_19_FN_3_O_4_^-^ | -3.1 | 225.0851 | C_14_H_10_FN_2_^-^ | 10.2 | | |
| M3^2a^ | | 9.66 | 386.1513 | ESIpos | Serum (2/2) | 0.006 | C_20_H_21_FN_3_O_4_^+^ | -0.8 | 253.0795 | C_15_H_10_FN_2_O^+^ | 7.1 | | |
| (*O*-demethyl, | |  |  |  |  |  |  |  | 109.0462 | C_7_H_6_F^+^ | 7.3 | | |
| OH-isopropyl) | |  | 384.1358 | ESIneg | Serum (2/2) | 0.007 | C_20_H_19_FN_3_O_4_^-^ | -0.5 | 225.0852 | C_14_H_10_FN_2_^-^ | 10.7 | | |
| M4^2a^ | | 10.16 | 414.1474 | ESIpos | Serum (2/2) | 0.032 | C_21_H_21_FN_3_O_5_^+^ | 2.2 | 253.0788 | C_15_H_10_FN_2_O^+^ | 4.3 | | |
| (COOH-isopropyl) | |  |  |  |  |  |  |  | 109.0457 | C_7_H_6_F^+^ | 2.8 | | |
| M5^2a^ | | 10.37 | 414.1474 | ESIpos | Serum (2/2) | 0.037 | C_21_H_21_FN_3_O_5_^+^ | -0.2 | 253.0784 | C_15_H_10_FN_2_O^+^ | 2.8 | | |
| (COOH-isopropyl) | |  |  |  |  |  |  |  | 109.0457 | C_7_H_6_F^+^ | 2.8 | | |
| M6^2b^ | | 10.71 | 368.1433 | ESIpos | Serum (1/2) | 0.009 | C_20_H_19_FN_3_O_3_^+^ | 6.2 | 253.0785 | C_15_H_10_FN_2_O^+^ | 3.2 | | |
| (Lactone formation) | |  |  |  |  |  |  |  | 109.0461 | C_7_H_6_F^+^ | 6.4 | | |
| M7^2a^ | | 8.70 | 276.1351 | ESIpos | Urine (1/1) | 1 | C_14_H_18_N_3_O_3_^+^ | 1.1 | 159.0549 | C_9_H_7_N_2_O^+^ | -5.7 | | |
| (*O*-demethyl, *N*-dealkyl, | |  |  |  |  |  |  |  |  |  |  | | |
| *N*-methyl) | |  |  |  |  |  |  |  |  |  |  | | |
| M8^2a^ | | 7.74 | 260.1031 | ESIneg | Serum / Urine | 0.005 / 1 | C_13_H_14_N_3_O_3_^-^ | -1.5 | 117.0455 | C_7_H_5_N_2_^-^ | 1.7 | | |
| (*O*-demethyl, *N*-dealkyl) | |  |  |  | (1/2) / (1/1) |  |  |  |  |  |  | | |
| *Identification confidence level according to Schymanski et al. (2014). | | | | | | | | | | | | | |
| **Table S2.** UHPLC-HRMS detection of ADB-FUBINACA and its metabolites. | | | | | | | | | | | | |  |
| **Compound*** | | **RT (min)** | ***m/z*** | **ESI** | **Matrix (prevalence)** | **Relative area** | **Elemental composition** | **Mass error (ppm)** | **Fragment ions (*m/z*)** | **Elemental composition** | **Mass error (ppm)** | | |
| ADB-FUBINACA^1^ | | 11.32 | 383.1891 | ESIpos | Serum (2/2) | 1 | C_21_H_24_FN_4_O_2_^+^ | 2.1 | 366.1617 | C_21_H_21_FN_3_O_2_^+^ | -0.3 | | |
|  | |  |  |  |  |  |  |  | 253.0777 | C_15_H_10_FN_2_O^+^ | 0.0 | | |
|  | |  |  |  |  |  |  |  | 109.0453 | C_7_H_6_F^+^ | -0.9 | | |
| M1^2a^ | | 9.79 | 399.1835 | ESIpos | Serum (2/2) | 0.941 | C_21_H_24_FN_4_O_3_^+^ | 0.8 | 382.1570 | C_21_H_21_FN_3_O_3_^+^ | 0.8 | | |
| (OH-tert-butyl) | |  |  |  |  |  |  |  | 253.0779 | C_15_H_10_FN_2_O^+^ | 0.8 | | |
|  | |  |  |  |  |  |  |  | 109.0454 | C_7_H_6_F^+^ | 0 | | |
|  | |  | 397.1711 | ESIneg | Serum (2/2) | 0.247 | C_21_H_22_FN_4_O_3_^-^ | 8.8 | 225.0843 | C_14_H_10_FN_2_^-^ | 6.7 | | |
| M2^2a^ | | 9.99 | 397.1686 | ESIpos | Serum (2/2) | 0.426 | C_21_H_22_FN_4_O_3_^+^ | 2.5 | 253.0778 | C_15_H_10_FN_2_O^+^ | 0.4 | | |
| (OH-tert-butyl, | |  |  |  |  |  |  |  | 109.0447 | C_7_H_6_F^+^ | -6.4 | | |
| cyclization) | |  |  |  |  |  |  |  |  |  |  | | |
| M3^2a^ | | 5.53 | 291.1463 | ESIpos | Serum (2/2) | 0.121 | C_14_H_19_N_4_O_3_^+^ | 2.1 | 274.1193 | C_14_H_16_N_3_O_3_^+^ | 0.4 | | |
| (OH-tert-butyl, | |  |  |  |  |  |  |  | 216.114 | C_12_H_14_N_3_O^+^ | 1.4 | | |
| *N*-dealkyl) | |  |  |  |  |  |  |  | 145.0404 | C_8_H_5_N_2_O^+^ | 0.7 | | |
|  | |  | 289.1292 | ESIneg | Serum (2/2) | 0.096 | C_14_H_17_N_4_O_3_^-^ | -3.1 | 117.0451 | C_7_H_5_N_2_^-^ | -1.7 | | |
| M4^2b^ | | 8.59 | 398.1505 | ESIpos | Serum (2/2) | 0.088 | C_21_H_21_FN_3_O_4_^+^ | -2.8 | 253.0782 | C_15_H_10_FN_2_O^+^ | 2.0 | | |
| (Amide hydrolysis, | |  |  |  |  |  |  |  | 109.0455 | C_7_H_6_F^+^ | 0.9 | | |
| OH-tert-butyl, cyclization) | |  |  |  |  |  |  |  |  |  |  | | |
| M5^2b^ | | 10.21 | 395.1532 | ESIpos | Serum (2/2) | 0.470 | C_21_H_20_FN_4_O_3_^+^ | 3.3 | 253.0784 | C_15_H_10_FN_2_O^+^ | 2.8 | | |
| (OH-tert-butyl, oxidation, | |  |  |  |  |  |  |  | 109.0460 | C_7_H_6_F^+^ | 5.5 | | |
| cyclization) | |  |  |  |  |  |  |  |  |  |  | | |
| M6^2b^ | | 6.45 | 287.1149 | ESIpos | Serum (2/2) | 0.253 | C_14_H_15_N_4_O_3_^+^ | 1.7 | 198.1037 | C_12_H_12_N_3_^+^ | 3.0 | | |
| (OH-tert-butyl, oxidation, | |  |  |  |  |  |  |  | 145.0394 | C_8_H_5_N_2_O^+^ | -5.5 | | |
| cyclization, *N*-dealkyl) | |  |  |  |  |  |  |  |  |  |  | | |
|  | |  |  |  |  |  |  |  |  |  |  | | |
| **Table S2 (cont).** UHPLC-HRMS detection of ADB-FUBINACA and its metabolites. | | | | | | | | | | | | |  |
| **Compound** | | **RT (min)** | ***m/z*** | **ESI** | **Matrix** | **Relative area** | **Elemental composition** | **Mass error (ppm)** | **Fragment ions (*m/z*)** | **Elemental composition** | **Mass error (ppm)** | | |
| M7^2b^ | | 7.83 | 275.1515 | ESIpos | Serum (2/2) | 0.994 | C_14_H_19_N_4_O_2_^+^ | 2.5 | 230.1290 | C_13_H_16_N_3_O^+^ | -1.3 | | |
| (*N*-dealkyl) | |  |  |  |  |  |  |  | 212.1192 | C_13_H_14_N_3_^+^ | 1.9 | | |
|  | |  |  |  |  |  |  |  | 145.0401 | C_8_H_5_N_2_O^+^ | -0.7 | | |
|  | |  | 273.1369 | ESIneg | Serum (2/2) | 1 | C_14_H_17_N_4_O_2_^-^ | 6.2 | 117.0455 | C_7_H_5_N_2_^-^ | 1.7 | | |
| *Identification confidence level according to Schymanski et al. (2014). | | | | | | | | | | | | | |

| **Table S3.** UHPLC-HRMS detection of SDB-005 and its metabolites. | | | | | | | | | | |  |  |
| --- | --- | --- | --- | --- | --- | --- | --- | --- | --- | --- | --- | --- |
| **Compound*** | **RT (min)** | ***m/z*** | **ESI** | **Matrix (Prevalence)** | **Relative area** | **Elemental composition** | **Mass error (ppm)** | **Fragment ions (*m/z*)** | **Elemental composition** | **Mass error (ppm)** | | |
| SDB-005 | 13.56 | n.d. | - | - |  | - | - | - | - | - | | |
| M1a^2b^ | 10.54 | 233.1286 | ESIpos | Serum/Urine | 0.552 / | C_13_H_17_N_2_O_2_^+^ | -1.7 | 215.1187 | C_13_H_15_N_2_O^+^ | 1.4 | | |
| (*O*-denaphthyl) |  |  |  | (2/2) / (1/1) | 0.045 |  |  | 145.0402 | C_8_H_5_N_2_O^+^ | 0.0 | | |
| M1b^2b^ | 11.00 | 233.129 | ESIpos | Serum (2/2) | 0.278 | C_13_H_17_N_2_O_2_^+^ | 0.0 | 145.0404 | C_8_H_5_N_2_O^+^ | 1.4 | | |
| (*O*-denaphthyl) |  | 231.1154 | ESIneg | Serum (2/2) | 0.002 | C_13_H_15_N_2_O_2_^-^ | 8.7 | 187.1278 | C_12_H_15_N_2_^-^ | 23.0 | | |
|  |  |  |  |  |  |  |  | 117.0469 | C_7_H_5_N_2_^-^ | 13.7 | | |
| M2^2b^ | 8.64 | 409.1624 | ESIpos | Serum/Urine | 1 / 1 | C_19_H_25_N_2_O_8_^+^ | 3.2 | 233.1288 | C_13_H_17_N_2_O_2_^+^ | -0.9 | | |
| (*O*-denaphthyl + |  |  |  | (2/2) / (1/1) |  |  |  | 215.1183 | C_13_H_15_N_2_O^+^ | -0.5 | | |
| glucuronidation) |  |  |  |  |  |  |  | 145.0399 | C_8_H_5_N_2_O^+^ | -2.1 | | |
|  |  | 407.1466 | ESIneg | Serum/Urine | 1 / 1 | C_19_H_23_N_2_O_8_^-^ | 2.9 | 231.1151 | C_13_H_15_N_2_O_2_^-^ | 7.4 | | |
|  |  |  |  | (2/2) / (1/1) |  |  |  | 187.1252 | C_12_H_15_N_2_^-^ | 9.1 | | |
|  |  |  |  |  |  |  |  | 175.0255 | C_6_H_7_O_6_^-^ | 6.9 | | |
| M3^3^ | 5.55 | 249.1256 | ESIpos | Urine (1/1) | 0.028 | C_13_H_17_N_2_O_3_^+^ | 6.8 | 213.1033 | C_13_H_13_N_2_O^+^ | 2.3 | | |
| (*O*-denaphthyl, |  |  |  |  |  |  |  | 145.0403 | C_8_H_5_N_2_O^+^ | 0.7 | | |
| OH-pentyl) |  |  |  |  |  |  |  |  |  |  | | |
| M4^3^ | 7.12 | 249.1238 | ESIpos | Urine (1/1) | 0.618 | C_13_H_17_N_2_O_3_^+^ | -0.4 | 231.1131 | C_13_H_15_N_2_O_2_^+^ | -1.3 | | |
| (*O*-denaphthyl, |  |  |  |  |  |  |  | 213.1028 | C_13_H_13_N_2_O^+^ | 0.0 | | |
| OH-pentyl) |  |  |  |  |  |  |  | 185.1085 | C_12_H_13_N_2_^+^ | 3.2 | | |
|  |  |  |  |  |  |  |  | 145.0406 | C_8_H_5_N_2_O^+^ | 2.8 | | |
|  |  | 247.1106 | ESIneg | Urine (1/1) | 0.194 | C_13_H_15_N_2_O_3_^-^ | 9.3 | 203.1204 | C_12_H_15_N_2_O^-^ | 9.8 | | |
|  |  |  |  |  |  |  |  | 117.0469 | C_7_H_5_N_2_^-^ | 13.7 | | |
| *Identification confidence level according to Schymanski et al. (2014). | | | | | | | | | | | |  |
| n.d.: not detected. | | | | | | | | | | | |  |

| **Table S4.** UHPLC-HRMS detection of APINACA and its metabolites | | | | | | | | | | | | | | | | | | | |
| --- | --- | --- | --- | --- | --- | --- | --- | --- | --- | --- | --- | --- | --- | --- | --- | --- | --- | --- | --- |
| **Compound*** | **RT (min)** |  | ***m/z*** |  | **ESI** |  | **Matrix (Prevalence)** |  | **Relative area** |  | **Elemental composition** |  | **Mass error (ppm)** |  | **Fragment ions (*m/z*)** |  | **Elemental composition** |  | **Mass error (ppm)** |
| APINACA^1^ | 15.18 |  | 366.2553 |  | ESIpos |  | Serum (1/2) |  | 1 |  | C_23_H_32_N_3_O^+^ |  | 2.2 |  | 135.1173 |  | C_10_H_15_^+^ |  | -0.7 |
| M1^2b^ | 12.89 |  | 382.2504 |  | ESIpos |  | Serum (1/2) |  | 0.230 |  | C_23_H_32_N_3_O_2_^+^ |  | 2.4 |  | 151.1127 |  | C_10_H_15_O^+^ |  | 2.6 |
| (OH-adamantyl) |  |  |  |  |  |  |  |  |  |  |  |  |  |  |  |  |  |  |  |
| M2^2b^ | 11.93 |  | 398.2447 |  | ESIpos |  | Serum (1/2) |  | 0.222 |  | C_23_H_32_N_3_O_3_^+^ |  | 0.8 |  | 215.1186 |  | C_13_H_15_N_2_O^+^ |  | 0.9 |
| (2OH-adamantyl) |  |  |  |  |  |  |  |  |  |  |  |  |  |  |  |  |  |  |  |
| M3^2b^ | 10.82 |  | 414.2388 |  | ESIpos |  | Serum (1/2) |  | 0.079 |  | C_23_H_32_N_3_O_4_^+^ |  | -1.2 |  | 232.1467 |  | C_13_H_18_N_3_O^+^ |  | 7.30 |
| (3OH-adamantyl) |  |  |  |  |  |  |  |  |  |  |  |  |  |  | 215.1198 |  | C_13_H_15_N_2_O^+^ |  | 6.5 |
|  |  |  |  |  |  |  |  |  |  |  |  |  |  |  | 145.0383 |  | C_8_H_5_N_2_O^+^ |  | -13.1 |
| M4^2b^ | 10.91 |  | 414.2388 |  | ESIpos |  | Serum (1/2) |  | 0.112 |  | C_23_H_32_N_3_O_4_^+^ |  | -1.2 |  | 215.1192 |  | C_13_H_15_N_2_O^+^ |  | 3.7 |
| (3OH-adamantyl) |  |  |  |  |  |  |  |  |  |  |  |  |  |  | 145.0383 |  | C_8_H_5_N_2_O^+^ |  | -13.1 |
| M5^2b^ | 8.73 |  | 414.2399 |  | ESIpos |  | Urine (1/2) |  | 0.350 |  | C_23_H_32_N_3_O_4_^+^ |  | 1.4 |  | 230.1303 |  | C_13_H_16_N_3_O^+^ |  | 4.3 |
| (2OH-adamantyl + |  |  |  |  |  |  |  |  |  |  |  |  |  |  | 213.1033 |  | C_13_H_13_N_2_O^+^ |  | 2.3 |
| OH-pentyl) |  |  |  |  |  |  |  |  |  |  |  |  |  |  |  |  |  |  |  |
| M6^2b^ | 8.85 |  | 414.2403 |  | ESIpos |  | Urine (1/2) |  | 0.082 |  | C_23_H_32_N_3_O_4_^+^ |  | 2.4 |  | 248.1396 |  | C_13_H_18_N_3_O_2_^+^ |  | -1.2 |
| (2OH-adamantyl + |  |  |  |  |  |  |  |  |  |  |  |  |  |  | 213.1033 |  | C_13_H_13_N_2_O^+^ |  | 0.9 |
| OH-pentyl) |  |  |  |  |  |  |  |  |  |  |  |  |  |  |  |  |  |  |  |
| M7^2b^ | 7.52 |  | 430.2324 |  | ESIpos |  | Urine (1/2) |  | 1 |  | C_23_H_32_N_3_O_5_^+^ |  | -4.2 |  | 248.1400 |  | C_13_H_18_N_3_O_2_^+^ |  | 0.4 |
| (3OH-adamantyl + |  |  |  |  |  |  |  |  |  |  |  |  |  |  | 231.1131 |  | C_13_H_15_N_2_O_2_^+^ |  | -1.3 |
| OH-pentyl) |  |  |  |  |  |  |  |  |  |  |  |  |  |  | 213.1029 |  | C_13_H_13_N_2_O^+^ |  | 0.5 |
| *Identification confidence level according to Schymanski et al. (2014). | | | | | | | | | | | | | | | | | | | |

| **Table S5.** UHPLC-HRMS detection of CUMYL-4CN-BINACA and its metabolites. | | | | | | | | | | |  |  |
| --- | --- | --- | --- | --- | --- | --- | --- | --- | --- | --- | --- | --- |
| **Compound*** | **RT (min)** | ***m/z*** | **ESI** | **Matrix (Prevalence)** | **Relative area** | **Elemental composition** | **Mass error (ppm)** | **Fragment ions (*m/z*)** | **Elemental composition** | **Mass error (ppm)** | |  |
| CUMYL-4CN-BINACA^1^ | 10.75 | 361.2029 | ESIpos | Serum (2/2) | 0.334 | C_22_H_25_N_4_O^+^ | 0.3 | 243.1243 | C_13_H_15_N_4_O^+^ | -1.2 | |  |
|  |  |  |  |  |  |  |  | 226.0975 | C_13_H_12_N_3_O^+^ | -2.2 | |  |
|  |  |  |  |  |  |  |  | 145.0397 | C_8_H_5_N_2_O^+^ | -3.4 | |  |
|  |  |  |  |  |  |  |  | 119.0862 | C_9_H_11_^+^ | 0.8 | |  |
| M1^2b^ | 9.26 | 391.1767 | ESIpos | Serum (2/2) | 0.125 | C_22_H_23_N_4_O_3_^+^ | -0.8 | 243.1246 | C_13_H_15_N_4_O^+^ | 0.0 | |  |
| (COOH-cumyl) |  |  |  |  |  |  |  | 226.0977 | C_13_H_12_N_3_O^+^ | -1.3 | |  |
|  |  |  |  |  |  |  |  | 145.0399 | C_8_H_5_N_2_O^+^ | -2.1 | |  |
|  |  | 389.1637 | ESIneg | Serum (2/2) | 0.015 | C_22_H_21_N_4_O_3_^-^ | 5.9 | 345.1756 | C_21_H_21_N_4_O^-^ | 11.9 | |  |
|  |  |  |  |  |  |  |  | 198.1036 | C_12_H_12_N_3_^-^ | 2.5 | |  |
|  |  |  |  |  |  |  |  | 117.0464 | C_7_H_5_N_2_^-^ | 9.4 | |  |
| M2^2a^ | 10.54 | 366.1815 | ESIpos | Serum (2/2) | 1 | C_21_H_24_N_3_O_3_^+^ | -0.8 | 231.0772 | C_12_H_11_N_2_O_3_^+^ | 0.9 | |  |
| (Decyanation to COOH) |  |  |  |  |  |  |  | 213.0661 | C_12_H_9_N_2_O_2_^+^ | -1.4 | |  |
|  |  |  |  |  |  |  |  | 145.0398 | C_8_H_5_N_2_O^+^ | -2.8 | |  |
|  |  |  |  |  |  |  |  | 119.0859 | C_9_H_11_^+^ | -1.7 | |  |
|  |  | 364.1666 | ESIneg | Serum (2/2) | 1 | C_21_H_22_N_3_O_3_^-^ | 1.4 | 278.1301 | C_17_H_16_N_3_O^-^ | 2.9 | |  |
|  |  |  |  |  |  |  |  | 117.0461 | C_7_H_5_N_2_^-^ | 6.8 | |  |
| M3^2b^ | 9.95 | 280.1448 | ESIpos | Serum (2/2) | 0.017 | C_17_H_18_N_3_O^+^ | -0.7 | 145.0397 | C_8_H_5_N_2_O^+^ | -3.4 | |  |
| (*N*-dealkyl) |  | 278.1298 | ESIneg | Serum (2/2) | 0.003 | C_17_H_16_N_3_O^-^ | 1.8 | 117.0461 | C_7_H_5_N_2_^-^ | 6.8 | |  |
| M4^3^ | 9.02 | 382.1764 | ESIpos | Serum (2/2) | 0.039 | C_21_H_24_N_3_O_4_^+^ | -0.8 | 231.0754 | C_12_H_11_N_2_O_3_^+^ | -6.9 | |  |
| (Decyanation to COOH, |  |  |  |  |  |  |  | 213.0676 | C_12_H_9_N_2_O_2_^+^ | 5.6 | |  |
| OH-cumyl) |  |  |  |  |  |  |  | 145.0411 | C_8_H_5_N_2_O^+^ | 6.2 | |  |
|  |  | 380.1620 | ESIneg | Serum (2/2) | 0.031 | C_21_H_22_N_3_O_4_^-^ | 2.6 | 294.1251 | C_17_H_16_N_3_O_2_^-^ | 2.7 | |  |
|  |  |  |  |  |  |  |  | 117.0456 | C_7_H_5_N_2_^-^ | 2.6 | |  |
| *Identification confidence level according to Schymanski et al. (2014). | | | | | | | | | | | | |

| **Table S6.** UHPLC-HRMS detection of 5F-AMB-PINACA and its metabolites. | | | | | | | | | | | | | | | | | | | | | | | | | | | | | | | | | | | | | |  |  |  |  |  |
| --- | --- | --- | --- | --- | --- | --- | --- | --- | --- | --- | --- | --- | --- | --- | --- | --- | --- | --- | --- | --- | --- | --- | --- | --- | --- | --- | --- | --- | --- | --- | --- | --- | --- | --- | --- | --- | --- | --- | --- | --- | --- | --- |
| **Compound*** | | | | **RT (min)** | | ***m/z*** | | | **ESI** | | **Matrix (Prevalence)** | | **Relative area** | | | | **Elemental composition** | | | | | **Mass error (ppm)** | | | | | **Fragment ions (*m/z*)** | | | | **Elemental composition** | | | | | **Mass error (ppm)** | | | | | |  |
| 5F-AMB-PINACA^1^ | | | | 11.60 | | 364.2043 | | | ESIpos | | Serum/Urine | | 0.176 / | | | | C_19_H_27_FN_3_O_3_^+^ | | | | | 1.9 | | | | | 304.1826 | | | | C_17_H_23_FN_3_O^+^ | | | | | 0.3 | | | | | |  |
|  | | | |  | |  | | |  | | (1/2) / (1/2) | | 0.026 | | | |  | | | | |  | | | | | 233.1093 | | | | C_13_H_14_FN_2_O^+^ | | | | | 1.3 | | | | | |  |
|  | | | |  | |  | | |  | |  | |  | | | |  | | | | |  | | | | | 213.1030 | | | | C_13_H_13_N_2_O^+^ | | | | | 0.9 | | | | | |  |
|  | | | |  | |  | | |  | |  | |  | | | |  | | | | |  | | | | | 145.0392 | | | | C_8_H_5_N_2_O^+^ | | | | | -6.9 | | | | | |  |
| M1^2a^ | | | | 10.75 | | 350.1882 | | | ESIpos | | Serum/Urine | | 1 / 0.465 | | | | C_18_H_25_FN_3_O_3_^+^ | | | | | 0.6 | | | | | 304.1826 | | | | C_17_H_23_FN_3_O^+^ | | | | | 0.3 | | | | | |  |
| (*O*-demethyl) | | | |  | |  | | |  | | (1/2) / (1/2) | |  | | | |  | | | | |  | | | | | 233.1086 | | | | C_13_H_14_FN_2_O^+^ | | | | | -1.7 | | | | | |  |
|  | | | |  | |  | | |  | |  | |  | | | |  | | | | |  | | | | | 213.1032 | | | | C_13_H_13_N_2_O^+^ | | | | | 1.9 | | | | | |  |
|  | | | |  | |  | | |  | |  | |  | | | |  | | | | |  | | | | | 145.0400 | | | | C_8_H_5_N_2_O^+^ | | | | | -1.4 | | | | | |  |
|  | | | |  | | 348.1729 | | | ESIneg | | Serum/Urine | | 1 / 0.097 | | | | C_18_H_23_FN_3_O_3_^-^ | | | | | 1.7 | | | | | 185.1087 | | | | C_12_H_13_N_2_^-^ | | | | | 4.3 | | | | | |  |
|  | | | |  | |  | | |  | | (1/2) / (1/2) | |  | | | |  | | | | |  | | | | | 117.0470 | | | | C_7_H_5_N_2_^-^ | | | | | 14.5 | | | | | |  |
| M2^3^ | | | | 8.80 | | 366.1835 | | | ESIpos | | Urine (1/2) | | 0.169 | | | | C_18_H_25_FN_3_O_4_^+^ | | | | | 1.6 | | | | | 320.1769 | | | | C_17_H_23_FN_3_O^+^ | | | | | -1.6 | | | | | |  |
| (*O*-demethyl, | | | |  | |  | | |  | |  | |  | | | |  | | | | |  | | | | | 249.1043 | | | | C_13_H_14_FN_2_O_2_^+^ | | | | | 1.6 | | | | | |  |
| OH-fluoropentyl) | | | |  | |  | | |  | |  | |  | | | |  | | | | |  | | | | | 145.0406 | | | | C_8_H_5_N_2_O^+^ | | | | | 2.8 | | | | | |  |
|  | | | |  | | 364.1680 | | | ESIneg | | Urine (1/2) | | 0.189 | | | | C_18_H_23_FN_3_O_4_^-^ | | | | | 1.9 | | | | | 216.1154 | | | | C_12_H_14_N_3_O^-^ | | | | | 7.9 | | | | | |  |
|  | | | |  | |  | | |  | |  | |  | | | |  | | | | |  | | | | | 131.0632 | | | | C_8_H_7_N_2_^-^ | | | | | 17.5 | | | | | |  |
| M3^3^ | | | | 8.90 | | 366.1834 | | | ESIpos | | Urine (1/2) | | 0.194 | | | | C_18_H_25_FN_3_O_4_^+^ | | | | | 1.4 | | | | | 320.1779 | | | | C_17_H_23_FN_3_O^+^ | | | | | 1.6 | | | | | |  |
| (*O*-demethyl, | | | |  | |  | | |  | |  | |  | | | |  | | | | |  | | | | | 249.1048 | | | | C_13_H_14_FN_2_O_2_^+^ | | | | | 3.6 | | | | | |  |
| OH-fluoropentyl) | | | |  | |  | | |  | |  | |  | | | |  | | | | |  | | | | | 145.0403 | | | | C_8_H_5_N_2_O^+^ | | | | | 0.7 | | | | | |  |
|  | | | |  | | 364.1681 | | | ESIneg | | Urine (1/2) | | 0.204 | | | | C_18_H_23_FN_3_O_4_^-^ | | | | | 2.2 | | | | | 216.1144 | | | | C_12_H_14_N_3_O^-^ | | | | | 3.2 | | | | | |  |
| M4^2a^ | | | | 9.07 | | 366.1837 | | | ESIpos | | Serum/Urine | | 0.120 / | | | | C_18_H_25_FN_3_O_4_^+^ | | | | | 2.2 | | | | | 233.1091 | | | | C_13_H_14_FN_2_O^+^ | | | | | 0.4 | | | | | |  |
| (*O*-demethyl, | | | |  | |  | | |  | | (1/2) / (1/2) | | 0.465 | | | |  | | | | |  | | | | | 213.1023 | | | | C_13_H_13_N_2_O^+^ | | | | | -2.3 | | | | | |  |
| OH-isopropyl) | | | |  | |  | | |  | |  | |  | | | |  | | | | |  | | | | | 145.0406 | | | | C_8_H_5_N_2_O^+^ | | | | | 2.8 | | | | | |  |
|  | | | |  | | 364.1674 | | | ESIneg | | Urine (1/2) | | 0.601 | | | | C_18_H_23_FN_3_O_4_^-^ | | | | |  | | | | | 346.1601 | | | | C_18_H_21_FN_3_O_3_^-^ | | | | | 9.8 | | | | | |  |
|  | | | |  | |  | | |  | |  | |  | | | |  | | | | |  | | | | | 205.1151 | | | | C_12_H_14_FN_2_^-^ | | | | | 4.9 | | | | | |  |
|  | | | |  | |  | | |  | |  | |  | | | |  | | | | |  | | | | | 185.1091 | | | | C_12_H_13_N_2_^-^ | | | | | 6.5 | | | | | |  |
| **Table S6 (cont).** UHPLC-HRMS detection of 5F-AMB-PINACA and its metabolites. | | | | | | | | | | | | | | | | | | | | | | | | | | | | | | | | | | | | | | | |  |  |  |
| **Compound*** | | | | **RT (min)** | | ***m/z*** | | | **ESI** | | **Matrix (Prevalence)** | | **Relative area** | | | | **Elemental composition** | | | | | **Mass error (ppm)** | | | | | **Fragment ions (*m/z*)** | | | | **Elemental composition** | | | | | **Mass error (ppm)** | | | | | |  |
| M5^2b^ | | | | 7.71 | | 262.1195 | | | ESIpos | | Serum/Urine | | 0.065 / | | | | C_13_H_16_N_3_O_3_^+^ | | | | | 1.1 | | | | | 216.1135 | | | | C_12_H_14_N_3_O^+^ | | | | | -0.9 | | | | | |  |
| (*O*-demethyl, | | | |  | |  | | |  | | (2/2) / (2/2) | | 0.236 | | | |  | | | | |  | | | | | 198.1037 | | | | C_12_H_12_N_3_^+^ | | | | | 3.0 | | | | | |  |
| *N*-dealkyl) | | | |  | |  | | |  | |  | |  | | | |  | | | | |  | | | | | 145.0401 | | | | C_8_H_5_N_2_O^+^ | | | | | -0.7 | | | | | |  |
|  | | | |  | | 260.1040 | | | ESIneg | | Serum/Urine | | 0.071 / | | | | C_13_H_14_N_3_O_3_^-^ | | | | | 1.9 | | | | | 117.0462 | | | | C_7_H_5_N_2_^-^ | | | | | 7.7 | | | | | |  |
|  | | | |  | |  | | |  | | (2/2) / (2/2) | | 0.259 | | | |  | | | | |  | | | | |  | | | |  | | | | |  | | | | | |  |
| M6^2b^ | | | | 9.62 | | 394.1782 | | | ESIpos | | Serum/Urine | | 0.109 / | | | | C_19_H_25_FN_3_O_5_^+^ | | | | | 1.0 | | | | | 233.1093 | | | | C_13_H_14_FN_2_O^+^ | | | | | 1.3 | | | | | |  |
| (COOH-isopropyl) | | | |  | |  | | |  | | (2/2) / (2/2) | | 0.105 | | | |  | | | | |  | | | | | 213.1029 | | | | C_13_H_13_N_2_O^+^ | | | | | 0.5 | | | | | |  |
|  | | | |  | |  | | |  | |  | |  | | | |  | | | | |  | | | | | 145.0401 | | | | C_8_H_5_N_2_O^+^ | | | | | -0.7 | | | | | |  |
|  | | | |  | | 392.1632 | | | ESIneg | | Urine (2/2) | | 0.026 | | | | C_19_H_23_FN_3_O_5_^-^ | | | | | 2.5 | | | | | 360.1383 | | | | C_18_H_19_FN_3_O_4_^-^ | | | | | 6.4 | | | | | |  |
|  | | | |  | |  | | |  | |  | |  | | | |  | | | | |  | | | | | 316.1463 | | | | C_17_H_19_FN_3_O_2_^-^ | | | | | 0.6 | | | | | |  |
|  | | | |  | |  | | |  | |  | |  | | | |  | | | | |  | | | | | 185.1100 | | | | C_12_H_13_N_2_^-^ | | | | | 11.3 | | | | | |  |
| M7^2b^ | | | | 9.87 | | 394.1782 | | | ESIpos | | Urine (1/2) | | 0.017 | | | | C_19_H_25_FN_3_O_5_^+^ | | | | | 6.8 | | | | | 233.1113 | | | | C_13_H_14_FN_2_O^+^ | | | | | 9.9 | | | | | |  |
| (COOH-isopropyl) | | | |  | |  | | |  | |  | |  | | | |  | | | | |  | | | | | 213.1020 | | | | C_13_H_13_N_2_O^+^ | | | | | -3.8 | | | | | |  |
|  | | | |  | |  | | |  | |  | |  | | | |  | | | | |  | | | | | 145.0395 | | | | C_8_H_5_N_2_O^+^ | | | | | -4.8 | | | | | |  |
|  | | | |  | | 392.1663 | | | ESIneg | | Urine (1/2) | | 0.003 | | | | C_19_H_23_FN_3_O_5_^-^ | | | | | 10.5 | | | | | 360.1380 | | | | C_18_H_19_FN_3_O_4_^-^ | | | | | 5.6 | | | | | |  |
|  | | | |  | |  | | |  | |  | |  | | | |  | | | | |  | | | | | 316.1471 | | | | C_17_H_19_FN_3_O_2_^-^ | | | | | 3.2 | | | | | |  |
| M8^2a^ | | | | 9.92 | | 376.1875 | | | ESIpos | | Serum/Urine | | 0.186 | | | | C_19_H_26_N_3_O_5_^+^ | | | | | 0.8 | | | | | 298.1577 | | | | C_17_H_20_N_3_O_2_^+^ | | | | | 7.0 | | | | | |  |
| (Defluorination to | | | |  | |  | | |  | | (2/2) / (2/2) | | 0.098 | | | |  | | | | |  | | | | | 245.0930 | | | | C_13_H_13_N_2_O_3_^+^ | | | | | 1.6 | | | | | |  |
| COOH) | | | |  | |  | | |  | |  | |  | | | |  | | | | |  | | | | | 227.0819 | | | | C_13_H_11_N_2_O_2_^+^ | | | | | -0.9 | | | | | |  |
|  | | | |  | |  | | |  | |  | |  | | | |  | | | | |  | | | | | 217.1001 | | | | C_12_H_13_N_2_O_2_^+^ | | | | | 11.1 | | | | | |  |
|  | | | |  | | 374.1731 | | | ESIneg | | Serum/Urine | | 0.046 / | | | | C_19_H_24_N_3_O_5_^-^ | | | | | 3.5 | | | | | 274.1203 | | | | C_14_H_16_N_3_O_3_^-^ | | | | | 4.0 | | | | | |  |
|  | | | |  | |  | | |  | | (2/2) / (2/2) | | 0.035 | | | |  | | | | |  | | | | |  | | | |  | | | | |  | | | | | |  |
|  | | |  | |  | | |  | |  | |  | |  | | | | |  | | | | |  | | | | |  | | | | |  | | | | | |  |  |  |
|  | | |  | |  | | |  | |  | |  | |  | | | | |  | | | | |  | | | | |  | | | | |  | | | | | |  |  |  |
|  | | |  | |  | | |  | |  | |  | | | |  | | | | |  | | | | |  | | | | | |  | | | | |  | |  |  |  |  |
|  | | **Table S6 (cont).** UHPLC-HRMS detection of 5F-AMB-PINACA and its metabolites. | | | | | | | | | | | | | | | | | | | | | | | | | | | | | | | | | | | | |  |  |  |  |
| **Compound*** | | | | **RT (min)** | | ***m/z*** | | | **ESI** | | **Matrix (Prevalence)** | | **Relative area** | | | | | **Elemental composition** | | | | | **Mass error (ppm)** | | | | | **Fragment ions (*m/z*)** | | | | | **Elemental composition** | | | | | **Mass error (ppm)** | | | | |
| M9^2a^ | | | | 9.28 | | 348.1933 | | | ESIpos | | Urine (1/2) | | 0.071 | | | | | C_18_H_26_N_3_O_4_^+^ | | | | | 2.9 | | | | | 302.1877 | | | | | C_17_H_24_N_3_O_2_^+^ | | | | | 2.6 | | | | |
| (*O*-demethyl, oxidative | | | |  | |  | | |  | |  | |  | | | | |  | | | | |  | | | | | 231.1135 | | | | | C_13_H_15_N_2_O_2_^+^ | | | | | 0.4 | | | | |
| defluorination) | | | |  | | 346.1771 | | | ESIneg | | Urine (1/2) | | 0.060 | | | | | C_18_H_24_N_3_O_4_^-^ | | | | | 1.2 | | | | | 216.1152 | | | | | C_12_H_14_N_3_O^-^ | | | | | 6.9 | | | | |
|  | | | |  | |  | | |  | |  | |  | | | | |  | | | | |  | | | | | 117.0462 | | | | | C_7_H_5_N_2_^-^ | | | | | 7.7 | | | | |
| M10^3^ | | | | 6.85 | | 382.1797 | | | ESIpos | | Urine (1/2) | | 1 | | | | | C_18_H_25_FN_3_O_5_^+^ | | | | | 5.0 | | | | | 249.1037 | | | | | C_13_H_14_FN_2_O_2_^+^ | | | | | -0.8 | | | | |
| (*O*-demethyl, | | | |  | |  | | |  | |  | |  | | | | |  | | | | |  | | | | | 231.0942 | | | | | C_13_H_12_FN_2_O^+^ | | | | | 3.5 | | | | |
| OH-isopropyl, | | | |  | |  | | |  | |  | |  | | | | |  | | | | |  | | | | | 175.0512 | | | | | C_9_H_7_N_2_O_2_^+^ | | | | | 2.3 | | | | |
| OH-fluoropentyl) | | | |  | |  | | |  | |  | |  | | | | |  | | | | |  | | | | | 145.0400 | | | | | C_8_H_5_N_2_O^+^ | | | | | -1.4 | | | | |
|  | | | |  | | 380.1616 | | | ESIneg | | Urine (1/2) | | 1 | | | | | C_18_H_23_FN_3_O_5_^-^ | | | | | -1.6 | | | | | 316.1672 | | | | | C_17_H_22_N_3_O_3_^-^ | | | | | 3.5 | | | | |
|  | | | |  | |  | | |  | |  | |  | | | | |  | | | | |  | | | | | 232.1089 | | | | | C_12_H_14_N_3_O_2_^-^ | | | | | 1.3 | | | | |
|  | | | |  | |  | | |  | |  | |  | | | | |  | | | | |  | | | | | 131.0611 | | | | | C_8_H_7_N_2_^-^ | | | | | 1.5 | | | | |
|  | | | |  | |  | | |  | |  | |  | | | | |  | | | | |  | | | | | 117.0460 | | | | | C_7_H_5_N_2_^-^ | | | | | 6.0 | | | | |
| M11^3^ | | | | 6.97 | | 382.1800 | | | ESIpos | | Urine (1/2) | | 0.346 | | | | | C_18_H_25_FN_3_O_5_^+^ | | | | | 5.8 | | | | | 249.1037 | | | | | C_13_H_14_FN_2_O_2_^+^ | | | | | 1.2 | | | | |
| (*O*-demethyl, | | | |  | |  | | |  | |  | |  | | | | |  | | | | |  | | | | | 229.0989 | | | | | C_13_H_13_N_2_O_2_^+^ | | | | | 5.2 | | | | |
| OH-isopropyl, | | | |  | |  | | |  | |  | |  | | | | |  | | | | |  | | | | | 145.0405 | | | | | C_8_H_5_N_2_O^+^ | | | | | 2.1 | | | | |
| OH-fluoropentyl) | | | |  | | 380.1616 | | | ESIneg | | Urine (1/2) | | 0.336 | | | | | C_18_H_23_FN_3_O_5_^-^ | | | | | -1.6 | | | | | 232.1094 | | | | | C_12_H_14_N_3_O_2_^-^ | | | | | 3.4 | | | | |
|  | | | |  | |  | | |  | |  | |  | | | | |  | | | | |  | | | | | 131.0616 | | | | | C_8_H_7_N_2_^-^ | | | | | 5.3 | | | | |
|  | | | |  | |  | | |  | |  | |  | | | | |  | | | | |  | | | | | 117.0462 | | | | | C_7_H_5_N_2_^-^ | | | | | 7.7 | | | | |
| M12^3^ | | | | 7.20 | | 380.1639 | | | ESIneg | | Urine (2/2) | | 0.042 | | | | | C_18_H_23_FN_3_O_5_^-^ | | | | | 4.5 | | | | | 131.0617 | | | | | C_8_H_7_N_2_^-^ | | | | | 6.1 | | | | |
| (*O*-demethyl, | | | |  | |  | | |  | |  | |  | | | | |  | | | | |  | | | | |  | | | | |  | | | | |  | | | | |
| OH-isopropyl, | | | |  | |  | | |  | |  | |  | | | | |  | | | | |  | | | | |  | | | | |  | | | | |  | | | | |
| OH-fluoropentyl) | | | |  | |  | | |  | |  | |  | | | | |  | | | | |  | | | | |  | | | | |  | | | | |  | | | | |
|  | | |  | |  | | |  | |  | |  | | | |  | | | | |  | | | | |  | | | | | |  | | | | |  | |  |  |  |  |
|  | | |  | |  | | |  | |  | |  | | | |  | | | | |  | | | | |  | | | | | |  | | | | |  | |  |  |  |  |
|  | | |  | |  | | |  | |  | |  | | | |  | | | | |  | | | | |  | | | | | |  | | | | |  | |  |  |  |  |
|  | **Table S6 (cont).** UHPLC-HRMS detection of 5F-AMB-PINACA and its metabolites. | | | | | | | | | | | | | | | | | | | | | | | | | | | | | | | | | | | | | |  |  |  |  |
| **Compound*** | | | **RT (min)** | | ***m/z*** | | | **ESI** | | **Matrix (Prevalence)** | | **Relative area** | | | **Elemental composition** | | | | | **Mass error (ppm)** | | | | | **Fragment ions (*m/z*)** | | | | | **Elemental composition** | | | | | **Mass error (ppm)** | | | | | |  |  |
| M13^3^ | | | 7.64 | | 410.1741 | | | ESIpos | | Urine (1/2) | | 0.070 | | | C_19_H_25_FN_3_O_6_^+^ | | | | | 3.4 | | | | | 249.1046 | | | | | C_13_H_14_FN_2_O_2_^+^ | | | | | 2.8 | | | | | |  |  |
| (COOH-isopropyl, | | |  | |  | | |  | |  | |  | | |  | | | | |  | | | | | 231.0961 | | | | | C_13_H_12_FN_2_O^+^ | | | | | 11.7 | | | | | |  |  |
| OH-fluoropentyl) | | |  | | 408.1581 | | | ESIneg | | Urine (1/2) | | 0.042 | | | C_19_H_23_FN_3_O_6_^-^ | | | | | 2.5 | | | | | 184.0888 | | | | | C_11_H_10_N_3_^-^ | | | | | 7.1 | | | | | |  |  |
| M14^3^ | | | 7.84 | | 410.1744 | | ESIpos | | | Urine (2/2) | | 0.038 | | | C_19_H_25_FN_3_O_6_^+^ | | | | | 4.1 | | | | | 249.1048 | | | | | C_13_H_14_FN_2_O_2_^+^ | | | | | 3.6 | | | | | |  |  |
| (COOH-isopropyl, | | |  | | 408.1578 | | ESIneg | | | Urine (2/2) | | 0.024 | | | C_19_H_23_FN_3_O_6_^-^ | | | | | 1.7 | | | | | 184.0887 | | | | | C_11_H_10_N_3_^-^ | | | | | 6.5 | | | | | |  |  |
| OH-fluoropentyl) | | |  | |  | |  | | |  | |  | | |  | | | | |  | | | | |  | | | | |  | | | | |  | | | | | |  |  |
| M15^2b^ | | | 7.43 | | 360.1565 | | ESIpos | | | Urine (2/2) | | 0.385 | | | C_18_H_22_N_3_O_5_^+^ | | | | | 1.7 | | | | | 245.0924 | | | | | C_13_H_13_N_2_O_3_^+^ | | | | | -0.8 | | | | | |  |  |
| (*O*-demethyl, | | |  | |  | |  | | |  | |  | | |  | | | | |  | | | | | 227.0822 | | | | | C_13_H_11_N_2_O_2_^+^ | | | | | 0.4 | | | | | |  |  |
| defluorination to | | |  | |  | |  | | |  | |  | | |  | | | | |  | | | | | 217.1003 | | | | | C_12_H_13_N_2_O_2_^+^ | | | | | 12 | | | | | |  |  |
| COOH, | | |  | | 358.1407 | | ESIneg | | | Urine (2/2) | | 0.063 | | | C_18_H_20_N_3_O_5_- | | | | | 1.1 | | | | | 258.0888 | | | | | C_13_H_12_N_3_O_3_- | | | | | 3.5 | | | | | |  |  |
| lactone formation) | | |  | |  | |  | | |  | |  | | |  | | | | |  | | | | | 214.0996 | | | | | C_12_H_12_N_3_O- | | | | | 7.5 | | | | | |  |  |
|  | | |  | |  | |  | | |  | |  | | |  | | | | |  | | | | | 117.0459 | | | | | C_7_H_5_N_2_- | | | | | 5.1 | | | | | |  |  |
| M16^3^ | | | 7.08 | | 364.1676 | | ESIpos | | | Urine (2/2) | | 0.351 | | | C_18_H_23_FN_3_O_4_^+^ | | | | | 0.8 | | | | | 249.1044 | | | | | C_13_H_14_FN_2_O_2_^+^ | | | | | 2.0 | | | | | |  |  |
| (*O*-demethyl, | | |  | |  | |  | | |  | |  | | |  | | | | |  | | | | | 229.0983 | | | | | C_13_H_13_N_2_O_2_^+^ | | | | | 2.6 | | | | | |  |  |
| OH-fluoropentyl, | | |  | |  | |  | | |  | |  | | |  | | | | |  | | | | | 145.0402 | | | | | C_8_H_5_N_2_O^+^ | | | | | 0.0 | | | | | |  |  |
| lactone formation) | | |  | |  | |  | | |  | |  | | |  | | | | |  | | | | |  | | | | |  | | | | |  | | | | | |  |  |
| M17^3^ | | | 7.33 | | 364.1678 | | ESIpos | | | Urine (2/2) | | 0.034 | | | C_18_H_23_FN_3_O_4_^+^ | | | | | 1.4 | | | | | 249.1040 | | | | | C_13_H_14_FN_2_O_2_^+^ | | | | | 0.4 | | | | | |  |  |
| (*O*-demethyl, | | |  | |  | |  | | |  | |  | | |  | | | | |  | | | | | 229.0997 | | | | | C_13_H_13_N_2_O_2_^+^ | | | | | 8.7 | | | | | |  |  |
| OH-fluoropentyl, | | |  | |  | |  | | |  | |  | | |  | | | | |  | | | | | 145.0410 | | | | | C_8_H_5_N_2_O^+^ | | | | | 5.5 | | | | | |  |  |
| lactone formation) | | |  | |  | |  | | |  | |  | | |  | | | | |  | | | | |  | | | | |  | | | | |  | | | | | |  |  |
| M18^3^ | | | 5.18 | | 278.1143 | | ESIpos | | | Serum/Urine | | 0.004 / | | | C_13_H_16_N_3_O_4_^+^ | | | | | 0.7 | | | | | 260.1035 | | | | | C_13_H_14_N_3_O_3_^+^ | | | | | 0.0 | | | | | |  |  |
| (*O*-demethyl, *N*-dealkyl | | |  | |  | |  | | | (2/2) / (2/2) | | 0.236 | | |  | | | | |  | | | | | 145.04 | | | | | C_8_H_5_N_2_O^+^ | | | | | -1.4 | | | | | |  |  |
| OH-isopropyl) | | |  | | 276.0988 | | ESIneg | | | Urine (2/2) | | 0.495 | | | C_13_H_14_N_3_O_4_^-^ | | | | | 1.4 | | | | | 117.0461 | | | | | C_7_H_5_N_2_^-^ | | | | | 6.8 | | | | | |  |  |
|  | | |  | |  | |  | | |  | |  | | | |  | | | | |  | | | | |  | | | | | |  | | | | |  | |  |  |  |  |
|  | | |  | |  | |  | | |  | |  | | | |  | | | | |  | | | | |  | | | | | |  | | | | |  | |  |  |  |  |
|  | **Table S6 (cont).** UHPLC-HRMS detection of 5F-AMB-PINACA and its metabolites. | | | | | | | | | | | | | | | | | | | | | | | | | | | | | | | | | | | | | |  |  |  |  |
| **Compound*** | | | **RT (min)** | | ***m/z*** | | **ESI** | | | **Matrix (Prevalence)** | | **Relative area** | | | **Elemental composition** | | | | | **Mass error (ppm)** | | | | | **Fragment ions (*m/z*)** | | | | | **Elemental composition** | | | | | **Mass error (ppm)** | | | | | |  |  |
| M19^3^ | | | 6.34 | | 278.1147 | | ESIpos | | | Urine (1/2) | | 0.039 | | | C_13_H_16_N_3_O_4_^+^ | | | | | 2.2 | | | | | 232.1100 | | | | | C_12_H_14_N_3_O_2_^+^ | | | | | 6.0 | | | | | |  |  |
| (*O*-demethyl, *N*-dealkyl, | | |  | |  | |  | | |  | |  | | |  | | | | |  | | | | | 214.0996 | | | | | C_12_H_12_N_3_O^+^ | | | | | 7.5 | | | | | |  |  |
| OH-indazole) | | |  | |  | |  | | |  | |  | | |  | | | | |  | | | | | 161.0358 | | | | | C_8_H_5_N_2_O_2_^+^ | | | | | 4.3 | | | | | |  |  |
|  | | |  | | 276.0996 | | ESIneg | | | Urine (1/2) | | 0.044 | | | C_13_H_14_N_3_O_4_^-^ | | | | | 4.3 | | | | | 133.0420 | | | | | C_7_H_5_N_2_O^-^ | | | | | 13.5 | | | | | |  |  |
| M20^3^ | | | 5.93 | | 380.1625 | | ESIpos | | | Urine (2/2) | | 0.253 | | | C_18_H_23_FN_3_O_5_^+^ | | | | | 0.8 | | | | | 265.0992 | | | | | C_13_H_14_FN_2_O_3_^+^ | | | | | 1.5 | | | | | |  |  |
| (*O*-demethyl, | | |  | |  | |  | | |  | |  | | |  | | | | |  | | | | | 145.0405 | | | | | C_8_H_5_N_2_O^+^ | | | | | 2.1 | | | | | |  |  |
| isopropyl lactone, | | |  | |  | |  | | |  | |  | | |  | | | | |  | | | | |  | | | | |  | | | | |  | | | | | |  |  |
| 2OH-fluoropentyl) | | |  | |  | |  | | |  | |  | | |  | | | | |  | | | | |  | | | | |  | | | | |  | | | | | |  |  |
| M21^2b^ | | | 6.70 | | 370.0711 | | ESIneg | | | Urine (1/2) | | 0.536 | | | C_14_H_16_N_3_O_7_S^-^ | | | | | 0.5 | | | | | 290.1150 | | | | | C_14_H_16_N_3_O_4_^-^ | | | | | 3.1 | | | | | |  |  |
| (*N*-dealkyl, | | |  | |  | |  | | |  | |  | | |  | | | | |  | | | | | 230.0938 | | | | | C_12_H_12_N_3_O_2_^-^ | | | | | 3.5 | | | | | |  |  |
| *O*-sulphate-indazole) | | |  | |  | |  | | |  | |  | | |  | | | | |  | | | | | 214.0995 | | | | | C_12_H_12_N_3_O^-^ | | | | | 7.0 | | | | | |  |  |
|  | | |  | |  | |  | | |  | |  | | |  | | | | |  | | | | | 133.0413 | | | | | C_7_H_5_N_2_O^-^ | | | | | 8.3 | | | | | |  |  |
| M22^2b^ | | | 3.74 | | 354.0402 | | ESIneg | | | Urine (1/2) | | 0.253 | | | C_13_H_12_N_3_O_7_S^-^ | | | | | 1.7 | | | | | 274.0837 | | | | | C_13_H_12_N_3_O_4_- | | | | | 3.3 | | | | | |  |  |
| (*O*-demethyl, isopropyl | | |  | |  | |  | | |  | |  | | |  | | | | |  | | | | | 133.0421 | | | | | C_7_H_5_N_2_O- | | | | | 14.3 | | | | | |  |  |
| lactone, *N*-dealkyl | | |  | |  | |  | | |  | |  | | |  | | | | |  | | | | |  | | | | |  | | | | |  | | | | | |  |  |
| *O*-sulphate-indazole) | | |  | |  | |  | | |  | |  | | |  | | | | |  | | | | |  | | | | |  | | | | |  | | | | | |  |  |
| *Identification confidence level according to Schymanski et al. (2014). | | | | | | | | | | | | | | | | | | | | | | | | | | | | | | | | | | | | | | | | |  |  |

|  | **Table S7.** UHPLC-HRMS detection of 5F-AB-PINACA and its metabolites. | | | | | | | | | | | | | | | | |  |  |  |  |
| --- | --- | --- | --- | --- | --- | --- | --- | --- | --- | --- | --- | --- | --- | --- | --- | --- | --- | --- | --- | --- | --- |
| **Compound*** | | **RT (min)** | ***m/z*** | **ESI** | **Matrix (Prevalence)** | **Relative area** | **Elemental composition** | | **Mass error (ppm)** | | **Fragment ions (*m/z*)** | | **Elemental composition** | | | **Mass error (ppm)** | | | |  |  |
| 5F-AB-PINACA^1^ | | 10.05 | 349.2019 | ESIpos | Serum / Urine | 1 / 0.013 | C_18_H_26_FN_4_O_2_^+^ | | -6.0 | | 332.1773 | | C_18_H_23_FN_3_O_2_^+^ | | | -0.3 | | | |  |  |
|  | |  |  |  | (2/2) / (2/2) |  |  | |  | | 304.0916 | | C_17_H_23_FN_3_O^+^ | | | 0.3 | | | |  |  |
|  | |  |  |  |  |  |  | |  | | 233.1088 | | C_13_H_14_FN_2_O^+^ | | | -0.9 | | | |  |  |
|  | |  |  |  |  |  |  | |  | | 213.1027 | | C_13_H_13_N_2_O^+^ | | | -0.5 | | | |  |  |
|  | |  |  |  |  |  |  | |  | | 145.0401 | | C_8_H_5_N_2_O^+^ | | | -0.7 | | | |  |  |
| M1^3^ | | 6.83 | 365.1976 | ESIpos | Urine | 0.028 | C_18_H_26_FN_4_O_3_^+^ | | -3.6 | | 348.1719 | | C_18_H_23_FN_3_O_3_^+^ | | | -1.1 | | | |  |  |
| (OH-indazole) | |  |  |  | (1/2) |  |  | |  | | 320.1770 | | C_17_H_23_FN_3_O_2_^+^ | | | -1.2 | | | |  |  |
|  | |  |  |  |  |  |  | |  | | 229.0977 | | C_13_H_13_N_2_O_2_^+^ | | | 0.0 | | | |  |  |
|  | |  |  |  |  |  |  | |  | | 161.0350 | | C_8_H_5_N_2_O_2_^+^ | | | -0.6 | | | |  |  |
| M2^3^ | | 7.95 | 365.1994 | ESIpos | Serum / Urine | 0.333 / | C_18_H_26_FN_4_O_3_^+^ | | 1.4 | | 348.1734 | | C_18_H_23_FN_3_O_3_^+^ | | | 3.2 | | | |  |  |
| (OH-fluoropentyl) | |  |  |  | (2/2) / (2/2) | 1 |  | |  | | 320.1774 | | C_17_H_23_FN_3_O_2_^+^ | | | 0.0 | | | |  |  |
|  | |  |  |  |  |  |  | |  | | 249.1041 | | C_13_H_14_FN_2_O_2_^+^ | | | 0.8 | | | |  |  |
|  | |  |  |  |  |  |  | |  | | 231.0938 | | C_13_H_12_FN_2_O^+^ | | | 1.7 | | | |  |  |
|  | |  |  |  |  |  |  | |  | | 145.0399 | | C_8_H_5_N_2_O^+^ | | | -2.1 | | | |  |  |
|  | |  | 363.1862 | ESIneg | Serum / Urine | 0.308 / | C_18_H_24_FN_4_O_3_^-^ | | 8.3 | | 259.1194 | | C_13_H_15_N_4_O_2_^-^ | | | -0.4 | | | |  |  |
|  | |  |  |  | (2/2) / (2/2) | 0.342 |  | |  | | 141.0664 | | C_6_H_9_N_2_O_2_^-^ | | | 0.0 | | | |  |  |
| M3^3^ | | 8.09 | 365.1995 | ESIpos | Serum / Urine | 0.683 / | C_18_H_26_FN_4_O_3_^+^ | | 1.6 | | 348.1727 | | C_18_H_23_FN_3_O_3_^+^ | | | 1.1 | | | |  |  |
| (OH-fluoropentyl) | |  |  |  | (2/2) / (2/2) | 0.456 |  | |  | | 320.1774 | | C_17_H_23_FN_3_O_2_^+^ | | | 0.0 | | | |  |  |
|  | |  |  |  |  |  |  | |  | | 249.1037 | | C_13_H_14_FN_2_O_2_^+^ | | | -0.8 | | | |  |  |
|  | |  |  |  |  |  |  | |  | | 145.0404 | | C_8_H_5_N_2_O^+^ | | | 1.4 | | | |  |  |
|  | |  | 363.1830 | ESIneg | Serum / Urine | 0.780 / | C_18_H_24_FN_4_O_3_^-^ | | -0.6 | | 259.1197 | | C_13_H_15_N_4_O_2_^-^ | | | 0.8 | | | |  |  |
|  | |  |  |  | (2/2) / (2/2) | 0.199 |  | |  | | 141.0669 | | C_6_H_9_N_2_O_2_^-^ | | | 3.5 | | | |  |  |
|  | |  |  |  |  |  |  | |  | |  | |  | | |  | | | |  |  |
|  | |  |  |  |  |  |  | |  | |  | |  | | |  | | | |  |  |
|  | |  |  |  |  |  |  | |  | |  | |  | | |  | | | |  |  |
|  | |  |  |  |  |  |  | |  | |  | |  | | |  | | | |  |  |
|  | **Table S7 (cont).** UHPLC-HRMS detection of 5F-AB-PINACA and its metabolites. | | | | | | | | | | | | | | | | |  |  |  |  |
| **Compound*** | | **RT (min)** | ***m/z*** | **ESI** | **Matrix (Prevalence)** | **Relative area** | **Elemental composition** | | **Mass error (ppm)** | | **Fragment ions (*m/z*)** | | | **Elemental composition** | | | **Mass error (ppm)** | | | | |
| M4^3^ | | 8.25 | 365.1993 | ESIpos | Serum / Urine | 0.199 / | C_18_H_26_FN_4_O_3_^+^ | | 1.1 | | 348.1725 | | | C_18_H_23_FN_3_O_3_^+^ | | | 0.6 | | | | |
| (OH-fluoropentyl) | |  |  |  | (2/2) / (2/2) | 0.507 |  | |  | | 320.1779 | | | C_17_H_23_FN_3_O_2_^+^ | | | 1.6 | | | | |
|  | |  |  |  |  |  |  | |  | | 249.1037 | | | C_13_H_14_FN_2_O_2_^+^ | | | -0.8 | | | | |
|  | |  |  |  |  |  |  | |  | | 145.0396 | | | C_8_H_5_N_2_O^+^ | | | 0 | | | | |
|  | |  | 363.1827 | ESIneg | Serum / Urine | 0.221 / | C_18_H_24_FN_4_O_3_^-^ | | -1.4 | | 343.1775 | | | C_18_H_23_N_4_O_3_^-^ | | | 1.5 | | | | |
|  | |  |  |  | (2/2) / (2/2) | 0.266 |  | |  | | 201.1033 | | | C_12_H_13_N_2_O^-^ | | | 2.5 | | | | |
| M5^3^ | | 8.42 | 365.1998 | ESIpos | Serum / Urine | 0.174 / | C_18_H_26_FN_4_O_3_^+^ | | 2.5 | | 348.1746 | | | C_18_H_23_FN_3_O_3_^+^ | | | 6.6 | | | | |
| (OH-fluoropentyl) | |  |  |  | (2/2) / (2/2) | 0.135 |  | |  | | 320.1769 | | | C_17_H_23_FN_3_O_2_^+^ | | | -1.6 | | | | |
|  | |  |  |  |  |  |  | |  | | 249.1046 | | | C_13_H_14_FN_2_O_2_^+^ | | | 2.8 | | | | |
|  | |  |  |  |  |  |  | |  | | 145.0409 | | | C_8_H_5_N_2_O^+^ | | | 4.8 | | | | |
|  | |  | 363.1830 | ESIneg | Serum / Urine | 0. 225 / | C_18_H_24_FN_4_O_3_^-^ | | -0.6 | | 273.1354 | | | C_14_H_17_N_4_O_2_^-^ | | | 0.7 | | | | |
|  | |  |  |  | (2/2) / (2/2) | 0.082 |  | |  | | 141.0668 | | | C_6_H_9_N_2_O_2_^-^ | | | 2.8 | | | | |
|  | |  |  |  |  |  |  | |  | | 131.0616 | | | C_8_H_7_N_2_^-^ | | | 5.3 | | | | |
| M6^3^ | | 8.59 | 365.1993 | ESIpos | Urine (1/2) | 0.024 | C_18_H_26_FN_4_O_3_^+^ | | 1.1 | | 348.1723 | | | C_18_H_23_FN_3_O_3_^+^ | | | 1.7 | | | | |
| (OH-indazole) | |  |  |  |  |  |  | |  | | 320.1767 | | | C_17_H_23_FN_3_O_2_^+^ | | | -2.2 | | | | |
|  | |  |  |  |  |  |  | |  | | 249.1036 | | | C_13_H_14_FN_2_O_2_^+^ | | | -1.2 | | | | |
|  | |  |  |  |  |  |  | |  | | 229.0975 | | | C_13_H_13_N_2_O_2_^+^ | | | -0.9 | | | | |
|  | |  |  |  |  |  |  | |  | | 161.0350 | | | C_8_H_5_N_2_O_2_^+^ | | | -0.6 | | | | |
|  | |  | 363.1827 | ESIneg | Urine (1/2) | 0.053 | C_18_H_24_FN_4_O_3_^-^ | | -1.4 | | 318.1641 | | | C_17_H_21_N_3_O_2_F^-^ | | | 7.2 | | | | |
|  | |  |  |  |  |  |  | |  | | 221.1106 | | | C_12_H_14_N_2_OF^-^ | | | 7.2 | | | | |
|  | |  |  |  |  |  |  | |  | | 201.1041 | | | C_12_H_13_N_2_O^-^ | | | 6.5 | | | | |
| M7^3^ | | 8.69 | 365.1988 | ESIpos | Serum (2/2) | 0.235 | C_18_H_26_FN_4_O_3_^+^ | | -0.3 | | 348.1719 | | | C_18_H_23_FN_3_O_3_^+^ | | | -1.1 | | | | |
| (OH-isopropyl) | |  |  |  |  |  |  | |  | | 233.1079 | | | C_13_H_14_FN_2_O^+^ | | | -4.7 | | | | |
|  | |  |  |  |  |  |  | |  | | 213.1021 | | | C_13_H_13_N_2_O^+^ | | | -3.3 | | | | |
|  | |  |  |  |  |  |  | |  | | 145.0391 | | | C8H5N2O+ | | | -7.6 | | | | |
|  | |  |  |  |  |  |  | |  | |  | | |  | | |  | | | | |
|  | **Table S7 (cont).** UHPLC-HRMS detection of 5F-AB-PINACA and its metabolites. | | | | | | | | | | | | | | | | |  |  |  |  |
| **Compound*** | | **RT (min)** | ***m/z*** | **ESI** | **Matrix (Prevalence)** | **Relative area** | **Elemental composition** | | **Mass error (ppm)** | | **Fragment ions (*m/z*)** | | | **Elemental composition** | | | **Mass error (ppm)** | | | | |
| M7^3^ | |  | 363.1856 | ESIneg | Serum (2/2) | 1 | C_18_H_24_FN_4_O_3_^-^ | | -4.7 | | 185.1084 | | | C_12_H_13_N_2_^-^ | | | 13.0 | | | | |
| (OH-isopropyl) | |  |  |  |  |  |  | |  | | 157.0633 | | | C_6_H_9_N_2_O_3_^-^ | | | 12.1 | | | | |
|  | |  |  |  |  |  |  | |  | | 127.0510 | | | C_5_H_7_N_2_O_2_^-^ | | | 1.6 | | | | |
|  | |  |  |  |  |  |  | |  | | 112.0408 | | | C_5_H_6_NO_2_^-^ | | | 8.0 | | | | |
| M8^3^ | | 8.85 | 365.1998 | ESIpos | Serum / Urine | 0.235 / | C_18_H_26_FN_4_O_3_^+^ | | 2.5 | | 348.1731 | | | C_18_H_23_FN_3_O_3_^+^ | | | 2.3 | | | | |
| (OH-isopropyl) | |  |  |  | (2/2) / (2/2) | 0.011 |  | |  | | 233.1097 | | | C_13_H_14_FN_2_O^+^ | | | 3.0 | | | | |
|  | |  |  |  |  |  |  | |  | | 213.1033 | | | C_13_H_13_N_2_O^+^ | | | 2.3 | | | | |
|  | |  |  |  |  |  |  | |  | | 145.0398 | | | C_8_H_5_N_2_O^+^ | | | -2.8 | | | | |
| M9^2b^ | | 6.64 | 261.1352 | ESIpos | Serum / Urine | 0.179 / | C_13_H_17_N_4_O_2_^+^ | |  | | 216.1127 | | | C_12_H_14_N_3_O^+^ | | | -4.6 | | | | |
| (*N*-dealkyl) | |  |  |  | (2/2) / (2/2) | 0.011 |  | |  | | 198.1025 | | | C_12_H_12_N_3_^+^ | | | -3.0 | | | | |
|  | |  |  |  |  |  |  | |  | | 145.0399 | | | C_8_H_5_N_2_O^+^ | | | -2.1 | | | | |
|  | |  | 259.1200 | ESIneg | Serum / Urine | 0.795 / | C_13_H_15_N_4_O_2_^-^ | | 1.9 | | 117.0459 | | | C_7_H_5_N_2_^-^ | | | 5.1 | | | | |
|  | |  |  |  | (2/2) / (2/2) | 0.050 |  | |  | |  | | |  | | |  | | | | |
| M10^2a^ | | 10.75 | 350.1891 | ESIpos | Serum (2/2) | 0.042 | C_18_H_25_FN_3_O_3_^+^ | | 3.1 | | 233.1086 | | | C_13_H_14_FN_2_O^+^ | | | -1.7 | | | | |
| (Amide hydrolysis) | |  |  |  |  |  |  | |  | | 213.1055 | | | C_13_H_13_N_2_O^+^ | | | 12.7 | | | | |
| M11^2b^ | | 7.70 | 262.1192 | ESIpos | Serum / Urine | 0.070 / | C_13_H_16_N_3_O_3_^+^ | | 0.0 | | 216.1139 | | | C_12_H_14_N_3_O^+^ | | | 0.9 | | | | |
| (*N*-dealkyl, | |  |  |  | (2/2) / (2/2) | 0.051 |  | |  | | 198.1027 | | | C_12_H_12_N_3_^+^ | | | -2.0 | | | | |
| amide hydrolysis) | |  |  |  |  |  |  | |  | | 145.0397 | | | C_8_H_5_N_2_O^+^ | | | -3.4 | | | | |
|  | |  | 260.1034 | ESIneg | Serum / Urine | 0.128 / | C_13_H_14_N_3_O_3_^+^ | | -0.4 | | 216.1145 | | | C_12_H_14_N_3_O^+^ | | | 3.7 | | | | |
|  | |  |  |  | (2/2) / (2/2) | 0.053 |  | |  | | 117.0458 | | | C_7_H_5_N_2_^-^ | | | 4.3 | | | | |
| M12^3^ | | 6.44 | 381.1953 | ESIpos | Urine (2/2) | 0.050 | C_18_H_26_FN_4_O_4_^+^ | | 3.9 | | 364.1684 | | | C_18_H_23_FN_3_O_4_^+^ | | | 3.0 | | | | |
| (OH-fluoropentyl, | |  |  |  |  |  |  | |  | | 249.1043 | | | C_13_H_14_FN_2_O_2_^+^ | | | 1.6 | | | | |
| OH-isopropyl) | |  |  |  |  |  |  | |  | | 231.0938 | | | C_13_H_12_FN_2_O^+^ | | | 1.7 | | | | |
|  | |  |  |  |  |  |  | |  | | 145.0402 | | | C_8_H_5_N_2_O^+^ | | | 0 | | | | |
|  | |  |  |  |  |  |  | |  | |  | | |  | | |  | | | | |
|  | **Table S7 (cont).** UHPLC-HRMS detection of 5F-AB-PINACA and its metabolites. | | | | | | | | | | | | | | | | |  |  |  |  |
| **Compound*** | | **RT (min)** | ***m/z*** | **ESI** | **Matrix (Prevalence)** | **Relative area** | **Elemental composition** | | **Mass error (ppm)** | | **Fragment ions (*m/z*)** | | | **Elemental composition** | | | **Mass error (ppm)** | | | | |
| M12^3^ | |  | 379.1788 | ESIneg | Urine (2/2) | 0.064 | C_18_H_24_FN_4_O_4_^-^ | | 1.6 | | 359.1730 | | | C_18_H_23_N_4_O_4_^-^ | | | 3.1 | | | | |
| (OH-fluoropentyl, | |  |  |  |  |  |  | |  | | 257.1058 | | | C_13_H_13_N_4_O_2_^-^ | | | 7.4 | | | | |
| OH-isopropyl) | |  |  |  |  |  |  | |  | | 157.0631 | | | C_6_H_9_N_2_O_3_^-^ | | | 11.5 | | | | |
| M13^3^ | | 6.55 | 379.1788 | ESIneg | Urine (1/2) | 0.007 | C_18_H_24_FN_4_O_4_^-^ | | 1.6 | | 359.1745 | | | C_18_H_23_N_4_O_4_^-^ | | | 7.2 | | | | |
| (OH-fluoropentyl, | |  |  |  |  |  |  | |  | | 157.0618 | | | C_6_H_9_N_2_O_3_^-^ | | | 3.2 | | | | |
| OH-isopropyl) | |  |  |  |  |  |  | |  | |  | | |  | | |  | | | | |
| M14^3^ | | 6.67 | 381.1937 | ESIpos | Urine (2/2) | 0.200 | C_18_H_26_FN_4_O_4_^+^ | | -0.3 | | 364.1674 | | | C_18_H_23_FN_3_O_4_^+^ | | | 0.3 | | | | |
| (OH-fluoropentyl, | |  |  |  |  |  |  | |  | | 336.1722 | | | C_17_H_23_FN_3_O_3_^+^ | | | -0.3 | | | | |
| OH-isopropyl) | |  |  |  |  |  |  | |  | | 249.1037 | | | C_13_H_14_FN_2_O_2_^+^ | | | -0.8 | | | | |
|  | |  |  |  |  |  |  | |  | | 231.0936 | | | C_13_H_12_FN_2_O^+^ | | | 0.9 | | | | |
|  | |  |  |  |  |  |  | |  | | 145.0400 | | | C_8_H_5_N_2_O^+^ | | | -1.4 | | | | |
|  | |  | 379.1787 | ESIneg | Urine (2/2) | 0.042 | C_18_H_24_FN_4_O_4_^-^ | | 1.3 | | 359.1720 | | | C_18_H_23_N_4_O_4_^-^ | | | 0.3 | | | | |
|  | |  |  |  |  |  |  | |  | | 157.0630 | | | C_6_H_9_N_2_O_3_^-^ | | | 10.8 | | | | |
| M15^3^ | | 6.81 | 381.196 | ESIpos | Urine (2/2) | 0.231 | C_18_H_26_FN_4_O_4_^+^ | | 5.8 | | 336.1718 | | | C_17_H_23_FN_3_O_3_^+^ | | | -2.7 | | | | |
| (OH-fluoropentyl, | |  |  |  |  |  |  | |  | | 249.1034 | | | C_13_H_14_FN_2_O_2_^+^ | | | -2 | | | | |
| OH-isopropyl) | |  | 379.1800 | ESIneg | Urine (2/2) | 0.007 | C_18_H_24_FN_4_O_4_^-^ | | 4.7 | | 359.1727 | | | C_18_H_23_N_4_O_4_^-^ | | | 2.2 | | | | |
|  | |  |  |  |  |  |  | |  | | 157.0630 | | | C_6_H_9_N_2_O_3_^-^ | | | 10.8 | | | | |
| M16^3^ | | 6.91 | 381.1938 | ESIpos | Urine (2/2) | 0.093 | C_18_H_26_FN_4_O_4_^+^ | | 0.0 | | 364.1681 | | | C_18_H_23_FN_3_O_4_^+^ | | | 2.2 | | | | |
| (OH-fluoropentyl, | |  |  |  |  |  |  | |  | | 336.1721 | | | C_17_H_23_FN_3_O_3_^+^ | | | -0.6 | | | | |
| OH-isopropyl) | |  |  |  |  |  |  | |  | | 249.1037 | | | C_13_H_14_FN_2_O_2_^+^ | | | -0.8 | | | | |
|  | |  |  |  |  |  |  | |  | | 145.0399 | | | C_8_H_5_N_2_O^+^ | | | -2.1 | | | | |
| M17^3^ | | 7.05 | 381.1938 | ESIpos | Urine (2/2) | 0.027 | C_18_H_26_FN_4_O_4_^+^ | | 0.0 | | 336.1725 | | | C_17_H_23_FN_3_O_3_^+^ | | | 0.6 | | | | |
| (OH-fluoropentyl, | |  |  |  |  |  |  | |  | | 249.1042 | | | C_13_H_14_FN_2_O_2_^+^ | | | 1.2 | | | | |
| OH-isopropyl) | |  |  |  |  |  |  | |  | | 145.0400 | | | C_8_H_5_N_2_O^+^ | | | -1.4 | | | | |
|  | |  |  |  |  |  |  | |  | |  | | |  | | |  | | | | |
|  | **Table S7 (cont).** UHPLC-HRMS detection of 5F-AB-PINACA and its metabolites. | | | | | | | | | | | | | | | | |  |  |  |  |
| **Compound*** | | **RT (min)** | ***m/z*** | **ESI** | **Matrix (Prevalence)** | **Relative area** | **Elemental composition** | | **Mass error (ppm)** | | **Fragment ions (*m/z*)** | | | **Elemental composition** | | | **Mass error (ppm)** | | | | |
| M18^3^ | | 5.98 | 278.1145 | ESIpos | Urine (2/2) | 0.003 | C_13_H_16_N_3_O_4_^+^ | | 1.4 | | 232.1118 | | | C_12_H_14_N_3_O_2_^+^ | | | 13.8 | | | | |
| (Amide hydrolysis, | |  |  |  |  |  |  | |  | | 161.0355 | | | C_8_H_5_N_2_O_2_^+^ | | | 2.5 | | | | |
| *N*-dealkyl, OH-indazole) | |  | 276.0994 | ESIneg | Urine (2/2) | 0.002 | C_13_H_14_N_3_O_4_^-^ | | 3.6 | | 133.0418 | | | C_7_H_5_N_2_O^-^ | | | 12.0 | | | | |
| M19^3^ | | 6.30 | 278.1146 | ESIpos | Urine (2/2) | 0.017 | C_13_H_16_N_3_O_4_^+^ | | 1.8 | | 232.1096 | | | C_12_H_14_N_3_O_2_^+^ | | | 4.3 | | | | |
| (Amide hydrolysis, | |  |  |  |  |  |  | |  | | 161.0353 | | | C_8_H_5_N_2_O_2_^+^ | | | 1.2 | | | | |
| *N*-dealkyl, OH-indazole) | |  | 276.0991 | ESIneg | Urine (2/2) | 0.017 | C_13_H_14_N_3_O_4_^-^ | | 2.5 | | 133.0420 | | | C_7_H_5_N_2_O^-^ | | | 13.5 | | | | |
| M20^3^ | | 4.38 | 275.1167 | ESIneg | Urine (2/2) |  | C_13_H_15_N_4_O_3_^-^ | | 2.3 | | 117.0472 | | | C_7_H_5_N_2_^-^ | | | 16.2 | | | | |
| (*N*-dealkyl, OH-isopropyl) | |  |  |  |  |  |  | |  | |  | | |  | | |  | | | | |
| M21^3^ | | 4.59 | 277.1306 | ESIpos | Urine (2/2) | 0.003 | C_13_H_17_N_4_O_3_^+^ | | 1.8 | | 145.0401 | | | C_8_H_5_N_2_O^+^ | | | -0.7 | | | | |
| (*N*-dealkyl, OH-isopropyl) | |  | 275.1156 | ESIneg | Urine (2/2) | 0.015 | C_13_H_15_N_4_O_3_^-^ | | 4.4 | | 133.0409 | | | C_7_H_5_N_2_O^-^ | | | 5.3 | | | | |
|  | |  |  |  |  |  |  | |  | | 117.0468 | | | C_7_H_5_N_2_^-^ | | | 12.8 | | | | |
| M22^3^ | | 5.02 | 277.1309 | ESIpos | Urine (2/2) | 0.014 | C_13_H_17_N_4_O_3_^+^ | | 2.9 | | 232.1102 | | | C_12_H_14_N_3_O_2_^+^ | | | 6.9 | | | | |
| (*N*-dealkyl, OH-isopropyl) | |  |  |  |  |  |  | |  | | 145.0404 | | | C_8_H_5_N_2_O^+^ | | | 1.4 | | | | |
|  | |  | 275.1156 | ESIneg | Urine (2/2) | 0.029 | C_13_H_15_N_4_O_3_^-^ | | 4.4 | | 117.0468 | | | C_7_H_5_N_2_^-^ | | | 12.8 | | | | |
| M23^3^ | | 5.14 | 277.1319 | ESIpos | Urine (2/2) | 0.004 | C_13_H_17_N_4_O_3_^+^ | | 6.5 | | 232.1096 | | | C_12_H_14_N_3_O_2_^+^ | | | 4.3 | | | | |
| (*N*-dealkyl, OH-indazole) | |  |  |  |  |  |  | |  | | 161.0354 | | | C_8_H_5_N_2_O_2_^+^ | | | 1.9 | | | | |
|  | |  | 275.1155 | ESIneg | Urine (2/2) | 0.007 | C_13_H_15_N_4_O_3_^-^ | | 4.0 | | 117.0437 | | | C_7_H_5_N_2_^-^ | | | -13.7 | | | | |
| M24^2a^ | | 6.15 | 383.2088 | ESIpos | Urine (2/2) | 0.046 | C_18_H_28_FN_4_O_4_^+^ | | -1.8 | | 338.1868 | | | C_17_H_25_FN_3_O_3_^+^ | | | -3.5 | | | | |
| (Epoxide formation + | |  |  |  |  |  |  | |  | | 267.1141 | | | C_13_H_16_FN_2_O_3_^+^ | | | -1.5 | | | | |
| hydrolysis to dihydrodiol) | |  |  |  |  |  |  | |  | | 247.1075 | | | C_13_H_15_N_2_O_3_^+^ | | | -3.2 | | | | |
|  | |  | 381.1953 | ESIneg | Urine (2/2) | 0.008 | C_18_H_28_FN_4_O_4_^+^ | | 3.9 | | 141.0678 | | | C_6_H_9_N_2_O_2_^-^ | | | 9.9 | | | | |
|  | |  |  |  |  |  |  | |  | |  | | |  | | |  | | | | |
|  | |  |  |  |  |  |  | |  | |  | | |  | | |  | | | | |
|  | |  |  |  |  |  |  | |  | |  | | |  | | |  | | | | |
|  | |  |  |  |  |  |  | |  | |  | | |  | | |  | | | | |
|  | **Table S7 (cont).** UHPLC-HRMS detection of 5F-AB-PINACA and its metabolites. | | | | | | | | | | | | | | | | |  |  |  |  |
| **Compound*** | | **RT (min)** | ***m/z*** | **ESI** | **Matrix (Prevalence)** | **Relative area** | **Elemental composition** | | **Mass error (ppm)** | | **Fragment ions (*m/z*)** | | | **Elemental composition** | | | **Mass error (ppm)** | | | | |
| M25^3^ | | 7.13 | 361.1876 | ESIpos | Urine (1/2) | 0.013 | C_18_H_25_N_4_O_4_^+^ | | 0.0 | | 316.1669 | | | C_17_H_22_N_3_O_3_^+^ | | | 2.5 | | | | |
| (Oxidative | |  |  |  |  |  |  | |  | | 245.0926 | | | C_13_H_13_N_2_O_3_^+^ | | | 0.0 | | | | |
| defluorination, | |  |  |  |  |  |  | |  | |  | | |  | | |  | | | | |
| OH-fluoropentyl, | |  |  |  |  |  |  | |  | |  | | |  | | |  | | | | |
| oxidation to ketone) | |  |  |  |  |  |  | |  | |  | | |  | | |  | | | | |
| M26^3^ | | 7.46 | 361.1882 | ESIpos | Urine (1/2) | 0.005 | C_18_H_25_N_4_O_4_^+^ | | 1.7 | | 316.1699 | | | C_17_H_22_N_3_O_3_^+^ | | | 12.0 | | | | |
| (Oxidative | |  |  |  |  |  |  | |  | | 245.0931 | | | C_13_H_13_N_2_O_3_^+^ | | | 2.0 | | | | |
| defluorination, | |  |  |  |  |  |  | |  | |  | | |  | | |  | | | | |
| OH-fluoropentyl, | |  |  |  |  |  |  | |  | |  | | |  | | |  | | | | |
| oxidation to ketone) | |  |  |  |  |  |  | |  | |  | | |  | | |  | | | | |
| M27^3^ | | 7.83 | 361.1888 | ESIpos | Urine (1/2) | 0.014 | C_18_H_25_N_4_O_4_^+^ | | 3.3 | | 245.0938 | | | C_13_H_13_N_2_O_3_^+^ | | | 4.9 | | | | |
| (Oxidative | |  |  |  |  |  |  | |  | |  | | |  | | |  | | | | |
| defluorination, | |  |  |  |  |  |  | |  | |  | | |  | | |  | | | | |
| OH-fluoropentyl, | |  |  |  |  |  |  | |  | |  | | |  | | |  | | | | |
| oxidation to ketone) | |  |  |  |  |  |  | |  | |  | | |  | | |  | | | | |
| M28^2a^ | | 8.30 | 361.1877 | ESIpos | Urine (2/2) | 0.686 | C_18_H_25_N_4_O_4_^+^ | | 0.3 | | 316.1653 | | | C_17_H_22_N_3_O_3_^+^ | | | -2.5 | | | | |
| (Defluorination to COOH) | |  |  |  |  |  |  | |  | | 298.1553 | | | C_17_H_20_N_3_O_2_^+^ | | | -1.0 | | | | |
|  | |  |  |  |  |  |  | |  | | 245.0924 | | | C_13_H_13_N_2_O_3_^+^ | | | -0.8 | | | | |
|  | |  |  |  |  |  |  | |  | | 227.0818 | | | C_13_H_11_N_2_O_2_^+^ | | | -1.3 | | | | |
|  | |  |  |  |  |  |  | |  | | 217.0975 | | | C_12_H_13_N_2_O_2_^+^ | | | -0.9 | | | | |
|  | |  | 359.1713 | ESIneg | Urine (2/2) | 1 | C_18_H_23_N_4_O^4-^ | |  | | 259.1194 | | | C_13_H_15_N_4_O_2_^-^ | | | -0.4 | | | | |
|  | |  |  |  |  |  |  | |  | | 141.0665 | | | C_6_H_9_N_2_O_2_^-^ | | | 0.7 | | | | |
|  | |  |  |  |  |  |  | |  | | 117.0456 | | | C_7_H_5_N_2_^-^ | | | 2.6 | | | | |
|  | |  |  |  |  |  | |  | |  | |  | | |  | | | |  | |  |
|  | |  |  |  |  |  | |  | |  | |  | | |  | | | |  | |  |
|  | |  |  |  |  |  | |  | |  | |  | | |  | | | |  | |  |
|  | **Table S7 (cont).** UHPLC-HRMS detection of 5F-AB-PINACA and its metabolites. | | | | | | | | | | | | | | | | |  |  |  |  |
| **Compound*** | | **RT (min)** | ***m/z*** | **ESI** | **Matrix (Prevalence)** | **Relative area** | **Elemental composition** | | **Mass error (ppm)** | | **Fragment ions (*m/z*)** | | | **Elemental composition** | | | **Mass error (ppm)** | | | | |
| M29^3^ | | 7.07 | 377.1805 | ESIpos | Urine (1/2) | 0.014 | C_18_H_25_N_4_O_5_^+^ | | -5.3 | | 314.1507 | | | C_17_H_20_N_3_O_3_^+^ | | | 0.6 | | | | |
| (Defluorination to COOH, | |  | 375.1674 | ESIneg | Urine (1/2) | 0.024 | C_18_H_23_N4O_5_^-^ | | 1.6 | | 317.1268 | | | C_15_H_17_N_4_O_4_^-^ | | | 5.7 | | | | |
| OH-isopropyl) | |  |  |  |  |  |  | |  | |  | | |  | | |  | | | | |
| M30^2a^ | | 8.50 | 347.2088 | ESIpos | Urine (2/2) | 0.017 | C_18_H_27_N_4_O_3_^+^ | | 1.4 | | 330.1820 | | | C_18_H_24_N_3_O_3_^+^ | | | 0.6 | | | | |
| (Oxidative defluorination) | |  |  |  |  |  |  | |  | | 302.1866 | | | C_17_H_24_N_3_O_2_^+^ | | | -1.0 | | | | |
|  | |  |  |  |  |  |  | |  | | 231.1132 | | | C_13_H_15_N_2_O_2_^+^ | | | -0.9 | | | | |
|  | |  |  |  |  |  |  | |  | | 213.1025 | | | C_13_H_13_N_2_O^+^ | | | -1.4 | | | | |
| M31^3^ | | 7.73 | 361.1683 | ESIneg | Urine (1/2) | 0.009 | C_18_H_22_FN_4_O_3_^-^ | | 1.9 | | 199.0891 | | | C_12_H_11_N_2_O^-^ | | | 10.0 | | | | |
| (OH-fluoropentyl, oxidation to ketone) | |  |  |  |  |  |  | |  | |  | | |  | | |  | | | | |
| M32^3^ | | 4.08 | 355.0728 | ESIneg | Urine (2/2) | 0.025 | C_13_H_15_N_4_O_6_S^-^ | | 4.5 | | 275.1154 | | | C_13_H_15_N_4_O_3_^-^ | | | 3.6 | | | | |
| (*N*-dealkyl, | |  |  |  |  |  |  | |  | | 133.0420 | | | C_7_H_5_N_2_O^-^ | | | 13.5 | | | | |
| indazole *O*-sulphate) | |  |  |  |  |  |  | |  | |  | | |  | | |  | | | | |
| M33^3^ | | 4.60 | 355.0715 | ESIneg | Urine (2/2) | 0.100 | C_13_H_15_N_4_O_6_S^-^ | | 0.8 | | 275.1151 | | | C_13_H_15_N_4_O_3_^-^ | | | 2.5 | | | | |
| (*N*-dealkyl, | |  |  |  |  |  |  | |  | | 133.0409 | | | C_7_H_5_N_2_O^-^ | | | 5.3 | | | | |
| indazole *O*-sulphate) | |  |  |  |  |  |  | |  | |  | | |  | | |  | | | | |
| *Identification confidence level according to Schymanski et al. (2014). | | | | | | | | | | | | | | | | | | | | | |

|  | **Table S8.** UHPLC-HRMS detection of THJ-2201 and its metabolites | | | | | | | | | | |  |
| --- | --- | --- | --- | --- | --- | --- | --- | --- | --- | --- | --- | --- |
| **Compound*** | | **RT (min)** | ***m/z*** | **ESI** | **Matrix (Prevalence)** | **Relative area** | **Elemental composition** | **Mass error (ppm)** | **Fragment ions (*m/z*)** | **Elemental composition** | **Mass error (ppm)** | |
| THJ-2201^1^ | | 13.06 | 361.1724 | ESIpos | Serum (2/2) | 1 | C_23_H_22_FN_2_O^+^ | 2.2 | 233.1099 | C_13_H_14_FN_2_O^+^ | 3.9 | |
|  | |  |  |  |  |  |  |  | 213.1035 | C_13_H_13_N_2_O^+^ | 3.3 | |
|  | |  |  |  |  |  |  |  | 145.0404 | C_8_H_5_N_2_O^+^ | 1.4 | |
| M1^2a^ | | 11.51 | 373.1555 | ESIpos | Serum (2/2) | 0.452 | C_23_H_21_N_2_O_3_^+^ | 0.8 | 155.0503 | C_11_H_7_O^+^ | 3.9 | |
| (Defluorination to COOH) | |  | 371.1418 | ESIneg | Serum (2/2) | 1 | C_23_H_19_N_2_O_3_^-^ | 5.9 | 271.0887 | C_18_H_11_N_2_O^-^ | 5.9 | |
| M2^3^ | | 8.66 | 389.1508 | ESIpos | Urine (1/2) | 1 | C_23_H_21_N_2_O_4_^+^ | 1.8 | 227.0843 | C_13_H_11_N_2_O_2_^+^ | 9.7 | |
| (Defluorination to COOH, | |  |  |  |  |  |  |  | 171.0453 | C_11_H_7_O_2_^+^ | 4.1 | |
| OH-naphtyl) | |  |  |  |  |  |  |  |  |  |  | |
| M3^3^ | | 8.69 | 405.1454 | ESIneg | Urine (1/2) | 1 | C_23_H_21_N_2_O_5_^+^ | 1.0 | 305.0945 | C_18_H_13_N_2_O_3_^+^ | 6.2 | |
| (Defluorination to COOH, | |  |  |  |  |  |  |  | 287.0829 | C_18_H_11_N_2_O_2_^+^ | 2.8 | |
| 2OH-naphtyl) | |  |  |  |  |  |  |  |  |  |  | |
| *Identification confidence level according to Schymanski et al. (2014). | | | | | | | | | | | | |

**Figure S1.** Proposed metabolic pathway for AMB-FUBINACA. ^*^Indicates previously reported *in vitro* metabolites (Fabregat-Safont et al. 2022).

**Figure S2.** Proposed metabolic pathway for ADB-FUBINACA. ^*^Indicates previously reported *in vitro* metabolites (Kavanagh et al. 2017, Carlier et al. 2017).

**Figure S3.** Proposed metabolic pathway for SDB-005.

**Figure S4.** Proposed metabolic pathway for APINACA. ^*^Indicates previously reported *in vitro* metabolites (Gandhi et al. 2013).

**Figure S5.** Proposed metabolic pathway for CUMYL-4CN-BINACA. ^*^Indicates previously reported *in vitro* metabolites (Öztürk et al. 2018, Stalberga et al. 2023).

**Figure S6.** Proposed metabolic pathway for 5F-AMB-PINACA. ^*^Indicates previously reported *in vitro* metabolites (Andersson et al. 2016).

**Figure S7.** Proposed metabolic pathway for 5F-AB-PINACA. ^*^Indicates previously reported *in vitro* metabolites (Wohlfarth et al. 2015).

**Figure S8.** Proposed metabolic pathway for THJ-2201. ^*^Indicates previously reported *in vitro* metabolites (Diao et al. 2016a).
